# Toward Sustainable Rare Earth Element Production: Key Challenges in Techno-Economic, Life Cycle, and Social Impact Assessment

**DOI:** 10.1021/acssuschemeng.5c06147

**Published:** 2026-02-03

**Authors:** Adam Smerigan, Rui Shi

**Affiliations:** † Department of Chemical Engineering, 8082 The Pennsylvania State University, University Park, Pennsylvania 16802, United States; ‡ Lawrence Livermore National Lab, Livermore, California 94550, United States; § Institute of Energy and the Environment, The Pennsylvania State University, University Park, Pennsylvania 16802, United States

**Keywords:** rare earth elements (REE), lanthanides, life cycle assessment (LCA), techno-economic analysis (TEA), social impact assessment, systems analysis, uncertainty analysis

## Abstract

Rare earth elements (REEs) are 17 critical minerals used in many clean energy technologies like wind turbines and electric vehicles. Conventionally, we produce REEs from mining in a few, geopolitically restricted regions. Developing systems that utilize new technologies and unconventional feedstocks provides an opportunity to meet increasing demand while improving sustainability. Techno-economic analysis (TEA), life cycle assessment (LCA), and social LCA (sLCA) are commonly used tools to assess the sustainability performance of these systems. However, analyses of REE systems encounter challenges, including system scope, data availability, technology scale-up, and uncertainty. In the reviewed literature, systems served multiple functions beyond producing REEs, including circularizing production and waste remediation, leading to discrepancies in scope. Further, the instability of REE prices led to high uncertainty due to different revenue, costs, and impact allocation. Therefore, these analyses leave decision makers with an incomplete understanding of the current landscape of REE production, inhibiting intelligent and efficient identification of future research and policy goals. In this narrative review, we conducted a comprehensive overview of the literature, synthesized studies from each pillar of sustainability (economic, environmental, and social), highlighted the challenges and limitations in each field, and recommended directions for future work developing sustainable REE production systems.

## Graphical abstract

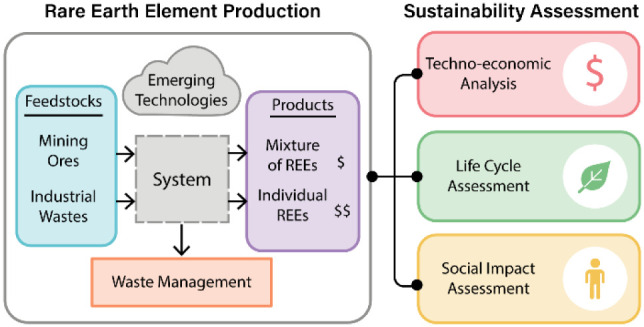

## 1 Introduction

To enable clean energy transitions and meet our climate goals,^1^ we must secure sustainable sources of critical minerals including rare earth elements (REEs). The demand for REEs (consisting of Sc, Y, and the lanthanides) is expected to increase by 3–7 times by 2040.^2^ This increase in production is unlikely to be filled by traditional mining efforts, which are both technically inefficient and geopolitically restricted. As a result, the future REE supply chain must integrate systems that utilize secondary sources using emerging technologies.^3^ However, the sustainability performance of these novel systems must be rigorously validated. Without such an assessment, there is a risk of investing in solutions that are unprofitable, environmentally harmful, or socially inequitable.

Currently, REEs are primarily produced through five production routes at specific production sites: Bayan Obo (BO, China), southern provinces (SP, China), Sichuan province (SC, China), Mount Weld (MW, Australia), and Mountain Pass (MP, United States). For Chinese production from bastnaesite/monazite ores (BO, SC) and ion adsorption clays (SP), the entire process (from mining through refining) is completed domestically. For production routes in Australia (primarily monazite) and the United States (bastnaesite), REEs from the ores are mined and extracted into a concentrated REE mixture before being shipped internationally (to Malaysia and China, respectively) for separation and refining. In total, China is estimated to produce 85% of unrefined REEs and 95% of all refined REEs.^4–6^ These current production routes have several negative aspects: geopolitical tensions affecting market stability, inefficient intra-REE separations,^7,8^ and impacts on local communities due to mining operations.^9^ Further, conventional mining may be unable to meet increased demand since opening additional mines can be a long and unsuccessful process.^10^ As a result, other production routes are being explored that use secondary REE feedstocks to reduce dependence on geographically concentrated virgin material mining, eliminate industrial wastes, and circularize the rare earth supply chain.

It is critical to assess the sustainability of these novel systems at early stages of development. To this end, several methods have been used to assess the environmental, economic, and social aspects of sustainability (LCA,^11,12^ TEA,^13–15^ and sLCA,^16,17^ respectively). Although prior reviews have examined these assessment methods for REE production systems,^18–21^ each has treated the environmental, economic, or social pillar in isolation, overlooking how methodological choices in one pillar directly influence conclusions in the others. Existing reviews rarely integrate qualitative social risk discussions with examination of the limited REE-focused sLCA literature and its associated methodological limitations. Likewise, the literature has not examined how decisions related to product and coproduct definitions propagate across TEA, LCA, and sLCA to shape system-level conclusions. For example, there is currently no established approach to compare the environmental impacts of mixed REE products generated by emerging technologies with the purified REE products reported in conventional LCA studies. Further, in the studies we identified, TEA results for unconventional REE production systems have not been consistently synthesized in a manner that enables cross-study comparison of how system scale, technology choice, coproduct strategies, and capital and operating costs influence profitability. Ultimately, the absence of cross-pillar integration has led to inconsistent assumptions and disconnected results, limiting the clarity and usefulness of these individual sustainability assessments. These shortcomings are often compounded by the limited uncertainty, sensitivity, and scenario analyses reported in the underlying studies (e.g., emphasis on local parameter variation and discrete scenarios rather than probabilistic sustainability assessment),^22^ which can hinder identification of key hotspots and the most influential parameters.

Without standardized and comprehensive sustainability analyses, we risk inefficient technology development and diminished incentives for innovative REE production systems—both of which are essential for meeting modern technological needs and supporting long-term climate and resource security. To advance a clearer understanding of the sustainability of emerging REE production pathways, this narrative review aims to (1) integrate knowledge across all three pillars of sustainability through a cross-cutting analysis, (2) provide an expanded qualitative and quantitative discussion of current methodological gaps, (3) supply practical resources to address these limitations, and (4) recommend methodological improvements for evaluating emerging REE production systems relative to conventional routes.

In this narrative review, we selected relevant studies using google scholar searches containing keywords related to the three pillars of sustainability for REEs within the title, abstract, and body of the main text. After synthesizing these studies and identifying the key knowledge gaps, we structured the main body of this text into sections to discuss the current landscape for REE production, the limitations of analyses for each pillar of sustainability, and our recommendations based on this review. These results will help researchers standardize sustainability assessment methodologies in the field and enable consistent comparisons between novel rare earth oxide (REO) production systems and conventional systems. Overall, this work will support the development of sustainable REE production systems that are economically viable, environmentally responsible, and designed to meet global needs while contributing to the circular economy.

## 2 Current Process Landscape for REE Recovery

Several flowsheets have been explored for secondary feedstocks including postconsumer products (e.g., catalysts, magnets), postindustrial waste (e.g., coal fly ash, acid mine drainage), and other minerals (e.g., lignite coal, phosphate rock).^23–25^ However, these feedstocks each have unique compositions of REEs and impurities that make REE extraction and purification challenging. Therefore, new technologies are being developed (e.g., bioleaching,^26,27^ electrochemical processes,^28,29^ sorptive separations^30,31^ ) to reduce the cost and environmental burden of these systems. Nevertheless, low technological readiness makes it difficult to assess the performance of these technologies at full-scale for comparison with conventional routes.^32,33^ Trying to use technological data from conventional REO production from REE containing ores is challenging since these data are proprietary.^34^ Nevertheless, certain information, including simple process flowsheets and cost data, is available in the literature.^20,34^ These simple flowsheets and fundamentals have been leveraged for alternative feedstocks. These routes generally follow Figure 1 where a solid feedstock is beneficiated followed by leaching of REEs into the liquid phase before removing impurities and selectively separating individual REEs for further refinement. Figure 1 also highlights some of the primary established and developing technologies used in REE production from a variety of feedstocks.

**Figure 1:**
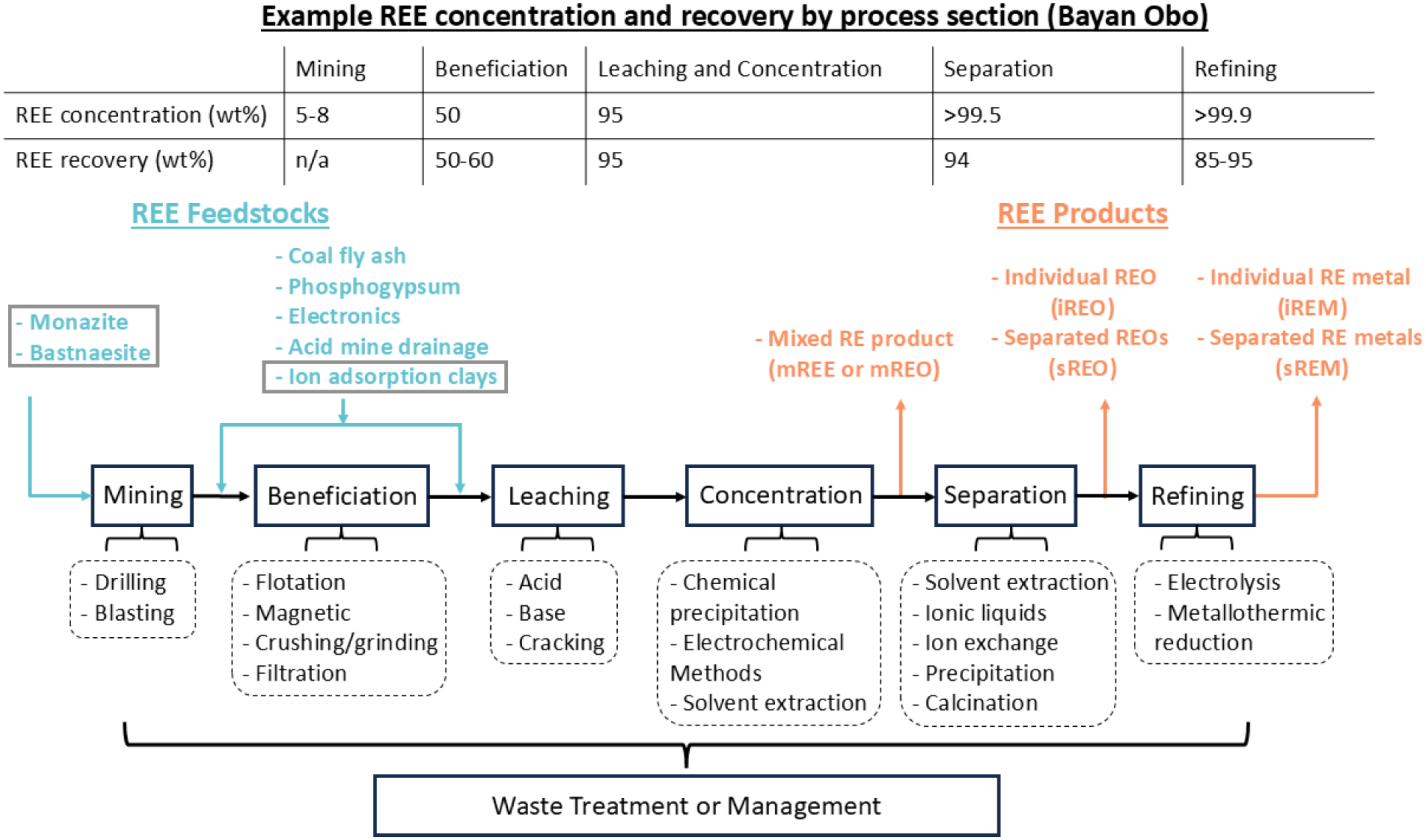
System diagram of rare earth element (REE) production from feedstock acquisition through refining. Different feedstocks are shown in blue text with conventional sources being boxed in gray. Process sections are shown in black boxes with potential technologies in dotted boxes. Different possible rare earth products are shown in orange text. Typical REE concentrations and recoveries out of each process section are shown in the table at the top using data from the Bayan Obo processing route.

From Figure 1, the production of REEs begins with mining, where drilling creates holes for explosives and blasting breaks the rock to access REE-bearing ores. The ore then undergoes beneficiation, which includes crushing and grinding the rock into fine particles, followed by flotation (using bubbles and chemicals to separate REE minerals), magnetic separation (pulling out magnetic minerals), and filtration (removing water and fine waste). Next comes leaching, in which acids, bases, or thermal-chemical “cracking” methods dissolve the REEs into solution. The dissolved REEs are then concentrated through techniques such as chemical precipitation (adding chemicals so that REEs form solid particles), electrochemical methods (e.g., electrodialysis, electrodeposition, electrocoagulation), or solvent extraction (using organic solvents to selectively dissolve REEs), which increase the proportion of REEs compared to impurities. After this, the individual REEs must be separated from each other, often using sequential solvent extraction stages, ionic liquids (special salts in liquid form that dissolve REEs), ion exchange resins. Purified REEs are then precipitated from solution (typically using oxalic acid) and are calcined into oxides. Finally, REOs can be further refined into pure REE metals by electrolysis, which uses electric current to reduce REE compounds, or by metallothermic reduction, which relies on reactive metals like calcium or magnesium to chemically reduce REE oxides. Other reviews have been published for specific feedstocks and technologies.^8,26,35^

Many unconventional feedstocks and technologies are currently being explored. Early stage systems analysis is essential to determine the economic, environmental and social trade-offs these processes and technologies have with those used in conventional REE processing routes.

### 2.1 Economics and Environmental Impact of Conventional REE Production

A previous study^20^ has compiled extensive economic data for conventional REE production processes. This review reported a large variance in the cost of operation for different REE production facilities. The capital expenditure (CAPEX) ranged from $0.016 to $608 per kilogram of REE produced (in 2022 US dollars) and the operational expenditure (OPEX) ranged from $0.91 to $11,000 per kilogram of REE produced. On average, REE production costs approximately $27.83·kg^–1^ REE. These costs are further broken down by process section with the REE-selective separation and refining comprising the majority of process costs (67% of the cost from mining through refining).^20^ The revenue of these REE production facilities is a function of the composition of the REEs in the feedstock as well as the REE purity. The price per kilogram of the sum of REEs produced is referred to as the basket price, which has varied widely over the past 20 years. A typical basket price has been reported to range from approximately $15 to $55·kg^–1^ REE, though higher basket prices were observed around 2011 due to policy changes in China.^36^

For the environmental impact of REE production, there have been many studies using a variety of different assumptions and uncertainties. Here, we summarize the reported global warming (GW) impact to quantify the collective uncertainty of several conventional REO production studies. The results for BO and SP have been multiplied by an adjustment factor of 1.19, calculated from literature data,^37^ that accounts for the extent and impact of illegal mining that occurs in these locations. Figure 2a shows that the GW impact varies nearly an order of magnitude across different studies for BO and SP routes. This high uncertainty makes it less clear whether novel systems have lower GW impact than conventional systems. Figure 2b shows how the GW contribution of process sections varies between the different conventional production routes. Additional details and the source of these literature data of conventional REE routes are provided in Table 2.^18,19,37–52^

**Figure 2:**
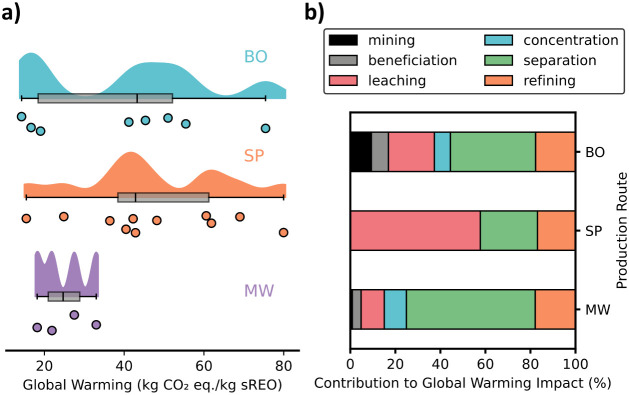
Compiled literature data for the global warming impact of conventional REE production routes (Bayan Obo China (BO), Southern Provinces China (SP), and Mount Weld Australia (MW)) per kilogram of separated REOs. This data is broken down into (a) the total global warming impact of each REE production route where each dot is a data point, the shaded regions are kernel density plots, and the gray is a boxplot showing the median, 1st and 3rd quartiles, and whiskers representing 1.5 times the interquartile range and (b) the average contribution of each process section to global warming impact.

Conventional REE production is both economically volatile and has substantial uncertainty in environmental footprint, with costs and emissions varying by orders of magnitude. These inconsistencies underscore the urgency of developing standardized metrics and benchmarks for evaluating alternative systems.

## 3 Limitations and Gaps in Present Sustainability Assessment Practices

### 3.1 Techno-Economic Analysis (TEA)

In the reviewed literature (Table 1), TEA studies investigated postindustrial waste (e.g., coal waste), the recycling of postconsumer wastes (e.g., NdFeB magnets), and ores. The REE content across feedstocks varied considerably from less than 0.001 wt % in acid mine drainage up to 50 wt % in monazite ore. Further, the most abundant REE varied by feedstock: Cerium (Ce) was the most prevalent in ores and postindustrial wastes (around 20–35 wt % REE), Neodymium (Nd) in NdFeB magnets (63–89 wt % REE) and cell phones (70 wt % REE), and Lanthanum (La) in fluid catalytic cracking (FCC) catalysts and NiMH batteries (97–100 wt %). In 16 of the 17 studies, the REE production capacity was at least 1 mt·year^–1^ with more abundant feedstocks having larger capacities. However, one study on NdFeB magnets^53^ considered a capacity less than 1 mt·year^–1^ leading to very unfavorable economics as compared to other NdFeB studies.^54–56^ Within these 17 studies, overall REE recovery varied (from 20 to 100 wt %) depending on feedstock composition and selected processing technologies. Depending on the feedstock, different contaminants and concentrations of REEs influenced leaching efficiency^53^ and solvent extraction performance.^20^ Overall, there is great diversity in the composition and availability of each feedstock leading to technological challenges and distinct design decisions. Additionally, there were inconsistencies in cost estimations regarding whether waste treatment and storage units are included in TEA.

**Table 1:** Summary of the TEA Studies Reviewed for Each Feedstock with Relevant Feedstock Information^a^

	Post-Consumer products						Post-Industrial waste				Mined ores	
	Fluid catalytic cracking (FCC) catalyst	NiMH batteries	Hard disk drive	Cell phone	NdFeB magnets	Lamp phosphors	Geothermal brine	Coal fly ash	Acid mine drainage (AMDp)	Phosphoric acid sludge, phosphogypsum	Monazite	Lignite coal
Reviewed studies (#)	1	1	1	1	4	1	1	2	3	2	1	1
References	^27^	^59^	^57^	^60^	^53–56^	^56^	^65^	^66,67^	^58,64,68^	^22,61^	^69^	^66^
Plant Capacity (mt/year)	18,800	2,000	342	2,000	0.33–2,270	2610–11,000	1,340	3,650–200,000	749–4,400,000	453,000–8,760,000	16,000	200,000
REE production (mt/year)	137	245	2.53	32	0.095–689	421–2,410	2.15	1.1–47.1	1–444	49–15,334	7,110–7,290	83
REE recovery (wt %)	28–56	85	75	78	60–100	66	90	45–75	80–93	17–48	80–90	75
REE content (wt %)	1.5	12	0.7–1	1.6	22–36	18–31	0.16	0.003–0.09	0.11–0.9	0.02–1.0	50	0.06
Primary REE (wt %)	La 97	La 100	PrNd 100	Nd 70	Nd 63–89	Y 90–92	Eu 100	Ce 33–36	Ce 25–26, Y 30	Ce 27–30	Ce 25	Ce 32
Basket Price ($/kg REO)	2–101	14	150	35	38–110	31–51	1038	22–104	24–104	51.5–256	14.5	491
Primary REE (% of revenue)	La 92–97	La 100	PrNd 100	Nd 92	Nd 36–82, Dy 51, Tb 84	Tb 97–99	Eu 100	Nd 37, Sc 70–90	Dy 18–23, Sc 80	Lu 73, Nd 47	Nd 50	Sc 70–94

a Feedstocks are grouped into 3 categories in the columns of the table. The rows of the table show the reviewed studies, plant capacity, REE recovery from the feedstock, REE content of the feedstock, the REE most prevalent in the feedstock (by mass and economic value), and the basket price of the REO Product

We summarize these TEA studies in Table 1 and discuss their results and limitations in five sections: 1) product identification, 2) product valuation, 3) operating costs (OPEX), 4) capital costs (CAPEX), and 5) profitability analysis. Here, we highlight some of the main points and variability in TEAs of REE systems, with methodological discussions following established TEA methodologies and industry practices.^14,33^ The TEAs reviewed here reveal high variability in product valuation, costs, and profitability, driven largely by feedstock composition and coproduct strategies. Consistent methodologies and transparency are critical to enable meaningful cross-study comparisons.

#### 3.1.1 Complexity in Product and Coproduct Identification

Table 1 shows the products across different feedstocks and system designs. Potential REE products include (1) mixed concentrates of REEs (mREE), which may occur as oxides (mREO), carbonates, mischmetal, or other compounds depending on the processing route; (2) individual REEs (iREE) in oxide, carbonate, or other forms; (3) reduced rare earth metals (REM); and (4) highly processed REE products (REEp), such as permanent magnets. Generally, products that are more pure have significantly improved value (with some variability due to the type of REE).^20^ However, further purification incurs additional costs. Therefore, potential incorporation of additional downstream processing must balance increasing revenue with cost. In 12 of the 17 studies, most of the revenue comes from only one REE, indicating that purification of the other REEs may not be worth the expense. Some studies have considered whether a mixed product has sufficient purity and composition to be directly used without incurring the expense of further separation (e.g., a Nd/Pr/Dy mixture for NdFeB magnets^54,57,58^ and a La/Ce mixture as mischmetal).^25^ Other studies have found that coproducts are the main source of revenue. These coproducts range from avoided waste tipping fees (FCC waste at 97% of revenue),^27^ other metal products (NiO at 79%^59^ and Au at 85%^60^ of revenue), and other chemicals (phosphoric acid at 71% of revenue).^61^ The revenue from these coproducts also helps insulate system profitability from the high variability of REE market values. Nevertheless, the identification and valuation of coproducts have been inconsistent across studies, and insufficient detail is reported regarding the value added from coproducts to determine optimal strategies.

#### 3.1.2 Limitations and Inconsistencies in REO/REE Product Valuation

Beyond identifying the REE products, it is challenging to estimate the value of each REE product. Many studies use the “basket price”, which evaluates the sREO value using the value of each individual REE within the feedstock.^36^ Basket prices were commonly reported in the literature for feedstocks containing multiple REEs (e.g., monazite, coal fly ash, acid mine drainage). For studies that did not explicitly list a basket price, we calculated the basket price to directly compare product values between feedstocks (Table 1). Basket prices varied widely from as little as $2/kg to over $400/kg in some cases. However, most basket prices varied between $50–150/kg. The high price ($1038/kg) for the geothermal brine study is due to producing solely a pure, high-value Eu product.

However, there are limitations to using the basket price. Basket prices are based on market data which is limited and highly variable (up to 400% fluctuation in REO values since 2010).^62^ Market prices are available primarily as >99.9% pure individual oxides (iREOs) or metals (REMs) making it difficult to assess the value of unpurified, mixed REE (mREE) products. To overcome this limitation, studies have used several methods to determine the value of REE products with varying accuracy. The least accurate method assumed iREO and mREE products have the same value. Another method applied discount factors to iREO values to estimate mREE values (assume the value of mREEs is around 20–60% the value of iREOs).^20,63^ Another method added a toll cost for paying an external (third party) solvent extraction process to convert their mREE product into an iREO product and iREO values were used to calculate revenue.^64^ Lastly, product value correlations were developed from available data to predict the value of REE products as their purity increases (from mREE to REEp).^20^ However, the REE balance problem may lead to deflation in the prices of more abundant REEs that are coproduced with desired, high-demand REEs.^3^ Therefore, operations with low operational expenses are expected to perform better than those with higher basket prices for REOs.^36^ In summary, there is high uncertainty in the value of REE products of varying purity making it difficult to quantify revenue and compare across different systems.

#### 3.1.3 Uncertainty and Variability in Operational Expense (OPEX)

From the literature reviewed, we broke down the OPEX into five distinct categories: materials, utilities, labor, maintenance, and other fixed costs (Figure 3). The cost of maintenance and other fixed costs were typically estimated as a percentage of other capital or operating costs. Labor costs were primarily determined by multiplying the number of shift-schedule workers who work rotating or nonstandard shifts (determined based on number of process sections and phase of materials) by salary. To calculate materials and utilities costs, several studies took prices from literature and online sources (e.g., Alibaba and mineralprices.com) and multiplied these prices by the rates of consumption for each item. However, prices listed on those websites can vary widely, lack verification, and may not reflect industrial-scale procurement costs. Using more standardized databases, such as the Independent Commodity Intelligence Services (ICIS) database,^70^ or supplier quotes would strengthen the quality of OPEX calculations and enable more consistent comparisons between studies. Prices from previous years were often converted to present value using inflation rates or price indices. Another key limitation observed across several studies is the lack of consideration of waste treatment or management. Of the 17 studies reviewed, only 8 mentioned these expenses in the text. Among these 8 studies, the reported costs either varied significantly or were not specified at all—some studies merely acknowledged their inclusion without providing exact figures.

**Figure 3:**
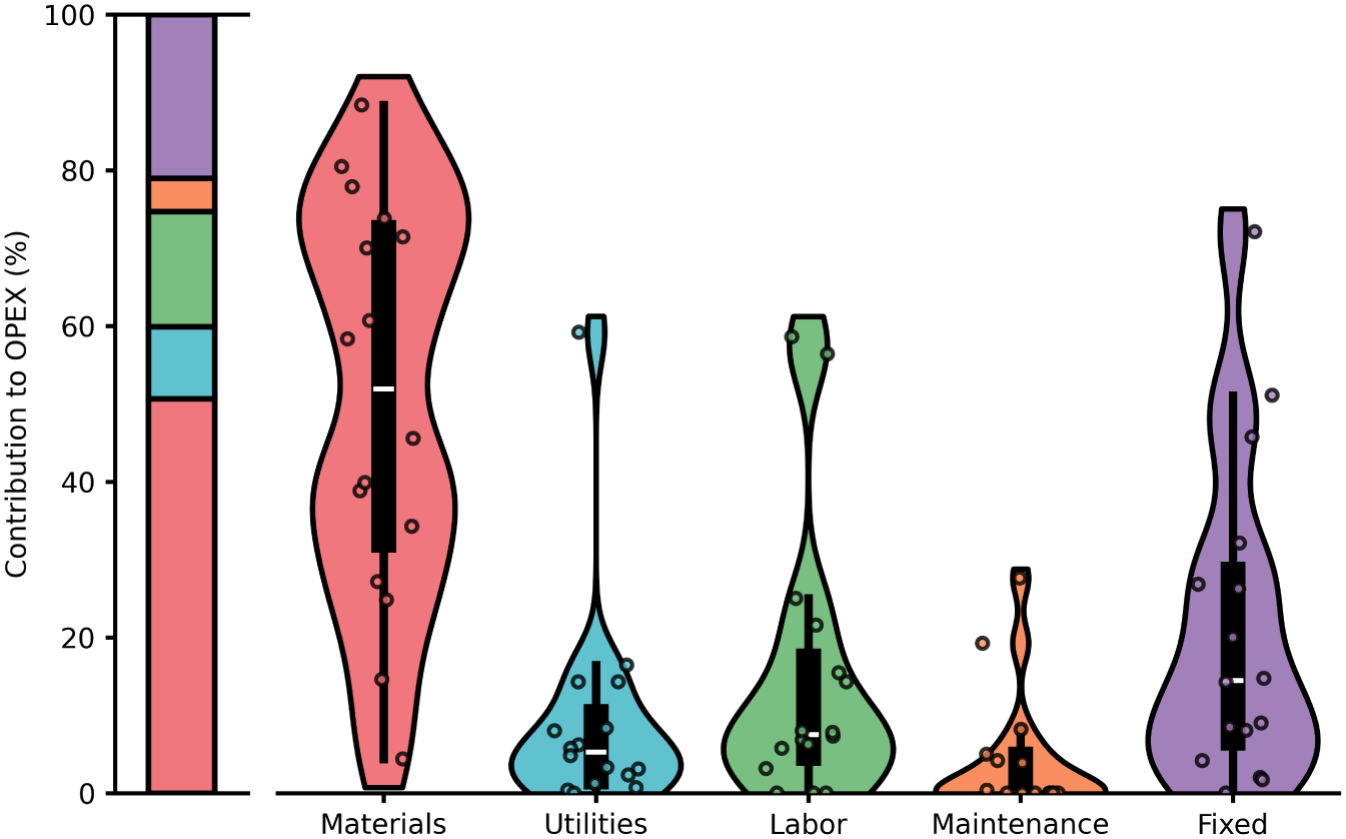
Contribution of different costs to total OPEX based on the average value from literature sources (left) and the variation of the values from these different sources (right) where each dot is a data point, the colored area is a kernel density distribution surrounding a box plot where the median is a white line.

Overall, material costs were the largest contributor to OPEX (mean of 51%) and had the largest variance (4–88%). This large variance is due to different feedstocks and technologies having different chemical requirements (e.g., whether acid leaching is required). The next largest contributor was other fixed costs (mean of 21%), which also had a large variance (0–72%). This variance is due to different methodologies for calculating fixed costs. Utilities, labor costs, and maintenance costs were least influential on the OPEX (9%, 15%, and 4%, respectively) and had less variation with only one or two outliers on the high end. For the studies with high labor costs, one paper^55^ was a batch process and the other paper^68^ gave no details on how the number was estimated. For the study with a high utility cost,^53^ high rates of material recycling reduced material costs leading to a higher contribution of utility costs to the total OPEX. Overall, material costs dominate in hydrometallurgical mining systems emphasizing the need for higher recycling rates and more efficient technologies that require fewer materials to extract and concentrate REE metals, whether through novel leaching technologies or more selective separations.

#### 3.1.4 Inconsistent and Inadequate Capital Expenses Estimation Methods for REE Systems

Capital expenditure (CAPEX) is the sum of main process area costs (e.g., inside battery limits costs like equipment purchase costs (EPC) and the foundations, piping, instrumentation, electrical, and installation labor for installing the equipment called installed costs), off-site and supporting infrastructure costs, engineering and construction costs, working capital, and contingency charges. For REE production systems in the literature, CAPEX estimation are performed using two methods: order-of-magnitude and study estimate methods (i.e., the Lang factor method). Using these methods, a TEA is expected to have ±50% and ±35% accuracy for the order-of-magnitude and study estimate methods, respectively.^14,71^ However, uncertainty can be as little as 5–10% as TRL increases and detailed purchase and installation cost estimates (e.g., vendor quotations) are obtained.

Beyond this inherent uncertainty, there is variability in the way CAPEX was estimated in the studies reviewed here. These studies used several methods to collect the EPC including literature sources, websites, and size factor calculations in process design textbooks. If purchase costs were obtained from smaller equipment, costs were scaled to size using the 6/10 rule, which is a commonly used engineering heuristic to estimate the change in equipment cost due to economies of scale.^14^ Other studies used size factors and process engineering textbook cost correlations to estimate full-scale equipment costs. It was unclear whether the EPC costs accounted for delivery costs. After calculating purchase costs, most studies converted prices that were obtained in past years to present day values using either an assumed inflation rate or cost indices (e.g., the Chemical Engineering Plant Cost Index, CEPCI). However, 8 of the 17 studies made no distinction as to the cost basis of their study. After calculating the present value of scaled up equipment, it was unclear how most studies calculated the installed cost of the equipment. Many studies mentioned using the Lang factor method but did not provide sufficient detail to understand what factor they used. The Lang factor method estimates total plant capital cost by multiplying the total purchased equipment cost by a literature-derived factor, which can vary by source. Other studies made no mention of how installed costs were calculated, if they were calculated at all. Beyond the process area costs, the extent to which “other investment costs” were estimated was highly variable. Of the 17 studies, 10 provided “other costs” (e.g., contingency or working capital) that were used in the final estimate of CAPEX. However, each study had its own list of “other costs” that ranged up to an order of magnitude between studies.

To better understand how CAPEX was estimated in these studies and to facilitate the standardization of methods in this emerging field, we calculated the ratio of total CAPEX to EPC. In Figure 4, these studies clustered into two distinct groups around ratios of 2 and 6. Most of the systems with high CAPEX:EPC ratios had similar REO production capacities and CAPEX to the other studies. These high ratio studies cited the process engineering textbook (e.g., Towler and Sinnott)^15^ for their CAPEX estimations suggesting that the recommended factors in these references may lead to higher CAPEX:EPC ratios. Higher CAPEX:EPC ratios agree more closely with generally accepted Lang factors which predict CAPEX:EPC ratios around 4–6 depending on whether the system handles predominantly fluids or solids.^14^ However, these Lang factors are based on decades-old data (the most up-to-date Lang factors available are from 2003)^13^ and may no longer be accurate, affecting the accuracy of CAPEX estimates. New developments in CAPEX estimation methods are needed to better reflect the characteristics of such emerging and novel processing.

**Figure 4:**
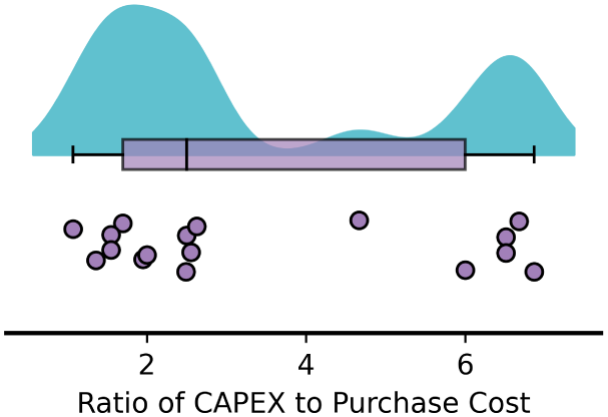
Ratio of capital expenditure to equipment purchase cost for each of the studies reviewed (purple dots). The statistics and distribution of the data are represented by a box plot (purple) and kernel density plot (blue).

#### 3.1.5 Transparency Issues in Profitability Evaluation and Lack of Discounted Cash Flow Analysis

To assess the economic viability of systems, economic indicators are compared between existing and proposed systems. Some indicators do not account for the time value of money (e.g., return on investment, payback period). Other indicators, such as net present value (NPV) and internal rate of return (IRR), consider the time value of money through a discounted cash flow (DCF) analysis. Of the 17 studies reviewed, 12 completed some form of DCF analysis, while the others reported only revenue and costs. For the calculation of NPV, studies used discount rates between 3 and 10% (8 of these 11 studies used rates between 8 and 10%). IRR was infrequently reported in the literature. Beyond discount rate, there are several other important parameters in DCF analysis. Studies considered plant lifetimes from 10 to 40 years with the average being between 20 and 30 years. Ten of the 17 studies included construction periods, and 5 studies included loan repayment schedules for CAPEX. Most studies included depreciation over 7–20 years using a variety of depreciation methods (e.g., straight line).

Of the 17 papers reviewed, 5 used an incomplete approach to profitability assessment by not including a DCF analysis, limiting both the accuracy of their evaluation and comparability across studies. For the studies reviewed, we directly report the NPV for studies that completed a DCF analysis. To compare across all studies, we calculated two metrics that do not include a DCF analysis: the return on investment (ROI) and cash flow (a common metric used in this field). The cash flow was calculated as the difference between annualized revenue and the annualized CAPEX and OPEX. Further, to consider a range of scenarios, we compiled the reported best- and worst-case values for given ranges of revenue and OPEX. For CAPEX, we used the average value for all scenarios to simplify the analysis since the range and magnitude of CAPEX values were small. Therefore, a study that gave ranges of revenue and OPEX would have 4 permutations in these results. These results in Figure 5a–c show that approximately 72% of systems were profitable across a range of different feedstocks and scenarios, regardless of whether the DCF analysis was completed. However, the return on investment (ROI) and cash flow metrics do not account for the time value of money. Therefore, these metrics are less useful for comparing between different investments over dissimilar project lifetimes.

**Figure 5:**
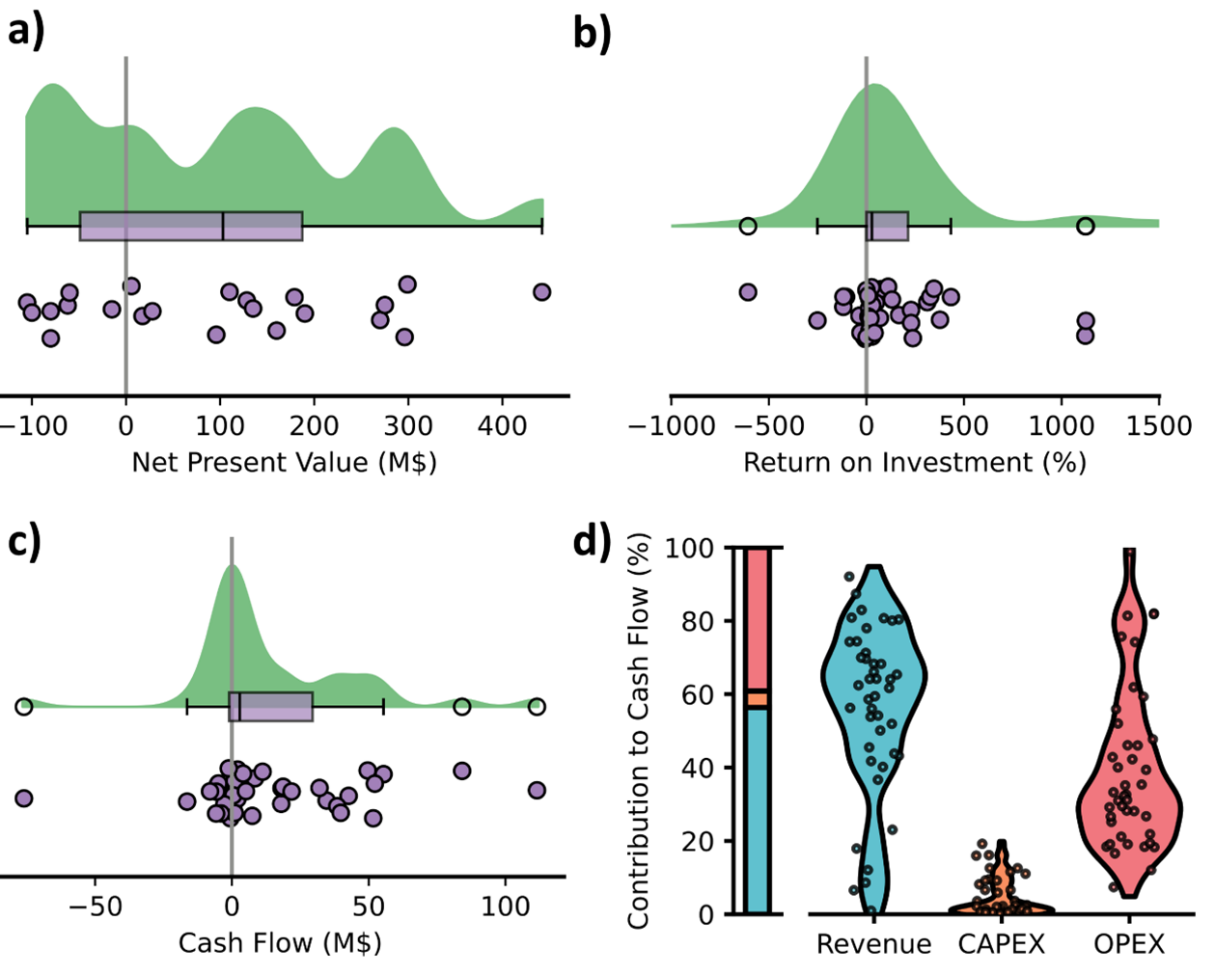
Profitability of REE production systems as (a) net present value, (b) return on investment, and (c) cash flow. (d) The total cash flow broken down into the contribution of annualized revenue, annualized CAPEX, and OPEX. The bar represents the mean of each contributor while the dots and violin plots show each sample and its distribution from the reviewed studies and scenarios.

To understand the main driver of profitability in these studies, we broke down the cash flows into revenue, annualized CAPEX, and OPEX for these systems (Figure 5d). We compiled this data across different revenue and OPEX scenarios to understand how different factors (e.g., prices, feedstock composition, technology efficiency, etc.) may affect profitability. Overall, the CAPEX had limited impact on the profitability of these systems. The OPEX contributed almost as much to the cash flow as revenue (36% and59%, respectively). This result suggests that systems with low OPEX will be more resilient to fluctuating REE values and are most likely to succeed, which agrees with other works.^20,36,64^ Unfortunately, achieving lower OPEX can be challenging for unconventional feedstocks with low REE content, as there is additional expense in extracting and concentrating these REEs. Therefore, technological innovations will be key for accessing low REE content feedstocks to compete with higher grade feedstocks.

### 3.2 Life Cycle Assessment (LCA)

To assess environmental impact, the most common method is life cycle assessment (LCA). LCA guidelines exist through international standards (ISO 14040 and 14044)^11,12^ making LCA more reputable, widely used, and comparable between studies. However, there are several issues limiting the reliability and usefulness of LCA for REO production technologies. Herein, we summarize the life cycle impacts of conventional REO production for assessing the life cycle environmental impact of novel REO production systems. We summarize the studies reviewed in Table 2.

**Table 2:** Summary of LCA Studies Reviewed including the Reference, Date of Publication, Feedstock, Functional Unit, LCIA Method, Allocation Method, and Co-Products Produced^a^

Feedstock^b^	Functional Unit	LCIA Method	Allocation^c^	Coproduct	Separate	Refine	Date	References
BO, SP	iREO, iREM, REEp	TRACI v2.1, ILCD	E	Iron ore, REOs	X	X	2018	^38^
BO, SP, MW	sREO	ILCD v1.09	E, S	Iron ore	X		2020	^18^
BO, MW, MP	REEp	ReCiPe v1.08, H	E, M	Iron ore, REOs	X	X	2018	^39^
BO	mREO	TRACI	E, M, O	Iron ore, REOs			2015	^40^
SP	mREO	Impact 2002+, USEtox 2.01, IPCC	-	-			2019	^41^
BO, SP	iREO, sREO	CML 2002	E, M	Iron ore, REOs	X	X	2017	^42^
BO, SP, SC	sREM	Eco-costs, others	-	Iron ore	X	X	2018	^37^
BO, SP, NK	iREO, sREO	ReCiPe v1.08, H	E, S	Iron ore, REOs	X	X	2018	^43^
all	iREO	n.a.	n.a.	Iron ore, REOs	X	X	2022	^44^
SP	mREO	TRACI, ILCD	E	-			2016	^45^
BO, SP	mREE	TRACI, ILCD	E	REOs	X		2017	^46^
SP	iREF, iREM	TRACI	E	REOs	X	X	2018	^47^
BO, NK	iREM	ReCiPe v1.08, H	E, S	Iron ore, REOs	X	X	2016	^48^
BO, SP	iREO, sREO	-	-	-	X		2021	^19^
BO	iREO	EI99	E, M	Iron ore, REOs	X		2014	^49^
MW	iREO, sREO	EI99, EI95	E	REOs	X		2020	^50^
SP	sREO	CML baseline v4.4	-	-	X		2017	^51^
BO, hard disk drives	REEp, sREO	CML 2001	E	Iron ore, REOs	X	X	2014	^52^
Idaho soil	Soil processed	CML-IA baseline	-	-			2024	^9^
Fluorescent lamp powder (FLP)	FLP processed	EF 3.0	D	mREO			2021	^72^
Phosphogypsum	PG treated	EI 99, H/A	D	Anhydrite, H3PO4			2016	^73^
Phosphogypsum	sREO, PG treated	ReCiPe 2016 H	-	Gypsum	X		2025	^22^
Coal refuse, lignite	mREO	TRACI v2.0	E	REOs			2020	^66^
Coal refuse, AMD	mREOH, sREO	TRACI	-	-	X		2024	^74^
NdFeB magnets	mREO	TRACI v2.1, CED	E	Iron oxide			2021	^54^
NdFeB magnets	REEp	TRACI	-	-			2016	^75^
e-waste	Gold	TRACI, ILCD	E	Silver, copper, mREO			2019	^76^
Hard disk drives	mREO	TRACI	E, M	Iron salt			2024	^57^
NdFeB magnets	REEp	EF v3.0	-	-		X	2024	^77^
LED waste	mREO, lighting service	ReCiPe H. end point	M, D	Mercury			2020	^78^
Fluid catalytic cracking (FCC) catalyst	FCC waste processed	TRACI v2.1	-	Disposal credit			2018	^27^
NdFeB magnets	mREO	CML-IA baseline v3.05	E, M	-			2022	^53^

a If mREEs are separated into sREO products, an “X” is marked in the “Separate” column. If sREOs are refined into sREM products, an “X” is marked in the “Refine” Column.

b Feedstocks for conventional mining are listed by their production route: monazite/bastnaesite at Bayan Obo (BO), ion adsorption clays in Southern Provinces, China (SP), bastnaesite at Mountain Pass, United States (MP), monazite at Mount Weld, Australia (MW), and eudialyte at Norra Kärr, Sweden (NK).

c Allocation methods used including economic (E), mass (M), other (O), subdivision (S), displacement (D).

Additionally, we break down the limitations of LCAs for systems producing REEs into several groups: 1) functional unit and scope, 2) multifunctionality and allocation, 3) life cycle inventory uncertainty, and 4) life cycle impact assessment uncertainty. Ideally, this work will bring to light some deficiencies of LCA studies for REO production and lead to more robust LCAs with clearer (or at least more informed) conclusions for decision makers.

#### 3.2.1 Functional Unit and System Boundary

Functional units are the foundational component of environmental assessments, providing a basis for quantifying the performance of different systems or products (an apples-to-apples comparison). However, within the 32 studies reviewed, there is a distinct lack of clarity for the specific product(s) being produced, which leads to confusion in interpreting the functional unit. One study goes as far as describing all REE compounds as REOs by stating the following: *“··· for the sake of clarity, we often refer to all of these different forms of rare earths as rare earth oxides (REO)*”.^52^ As we continue to advance sustainable solutions for REE recovery, we must carefully select functional units to enable meaningful comparisons across different approaches. There was very little variety in functional units chosen in the studies reviewed, with all studies reporting a functional unit of 1 kg (or metric ton) of some type of REE product produced. In Figure 1, we show a generic system diagram for REO production from primary and secondary sources, illustrating how different REE feedstocks enter the system at various process sections due to the varying extractability of REEs. Depending on the chosen scope of a study, five different products can be chosen as functional units of 1 kg of REE product produced (orange text). The first product exits the system after the concentration section as a mostly pure mixed REE product (mREE). This product can be a combination of multiple REOs (e.g., mischmetal, or other nonproduct composition) or REEs associated with another ion (e.g., RE carbonate or chloride). With further processing (e.g., solvent extraction), the mREEs can be separated into individual REEs. At this point, two different functional units can be defined: 1 kg of an individual purified REO produced (iREO, like Nd_2_O_3_) and 1 kg of separated REOs produced (sREO, the sum of iREOs after separation). This choice has implications for allocation discussed in Section 3.2.2. Further refinement of the sREOs through molten salt electrolysis or metallothermic reduction produces a pure rare earth metal (REM). As discussed above two functional units can be chosen for either 1 kg of individual (iREM) or 1 kg of separated rare earth metals (sREM, the sum of iREM produced) produced. Table 2 highlights the stark contrast in scope and functional unit between conventional routes and unconventional feedstocks. Specifically, looking at the separation column, only one study of an unconventional feedstock considers the selective intra-REE separation in the scope of the study illustrating that apples-to-apples comparisons to conventional systems is challenging.

To bridge this gap, we developed a method to enable comparisons between different functional units. In Figure 6, we provide an example using the GW impact of a mREE producing system to estimate the impact of separating and refining mREEs into sREO and sREM products, thereby converting between these functional units. The conversion from mREE to sREM requires four numbers: (1) the GW impact for a 100% pure mREE product (divide by purity if mREE has impurities), (2) the percentage of the original amount of REEs from the mREE in the final REE product (Figure 6d), (3) the fraction of impacts from the separation step (Figure 6e or Figure 2), and (4) a conversion to account for the mass of oxygen removed during refining. Figure 6b,c illustrates how to convert the “old”, or original, GW impact to the GW impact of the “new”, or desired, functional unit. This approach can be used to go from any functional unit to another, not just mREE to more purified product as demonstrated in this example. By developing approaches to compare systems with different functional units, we can enable rapid comparison of environmental impacts between systems to enhance decision-making.

**Figure 6:**
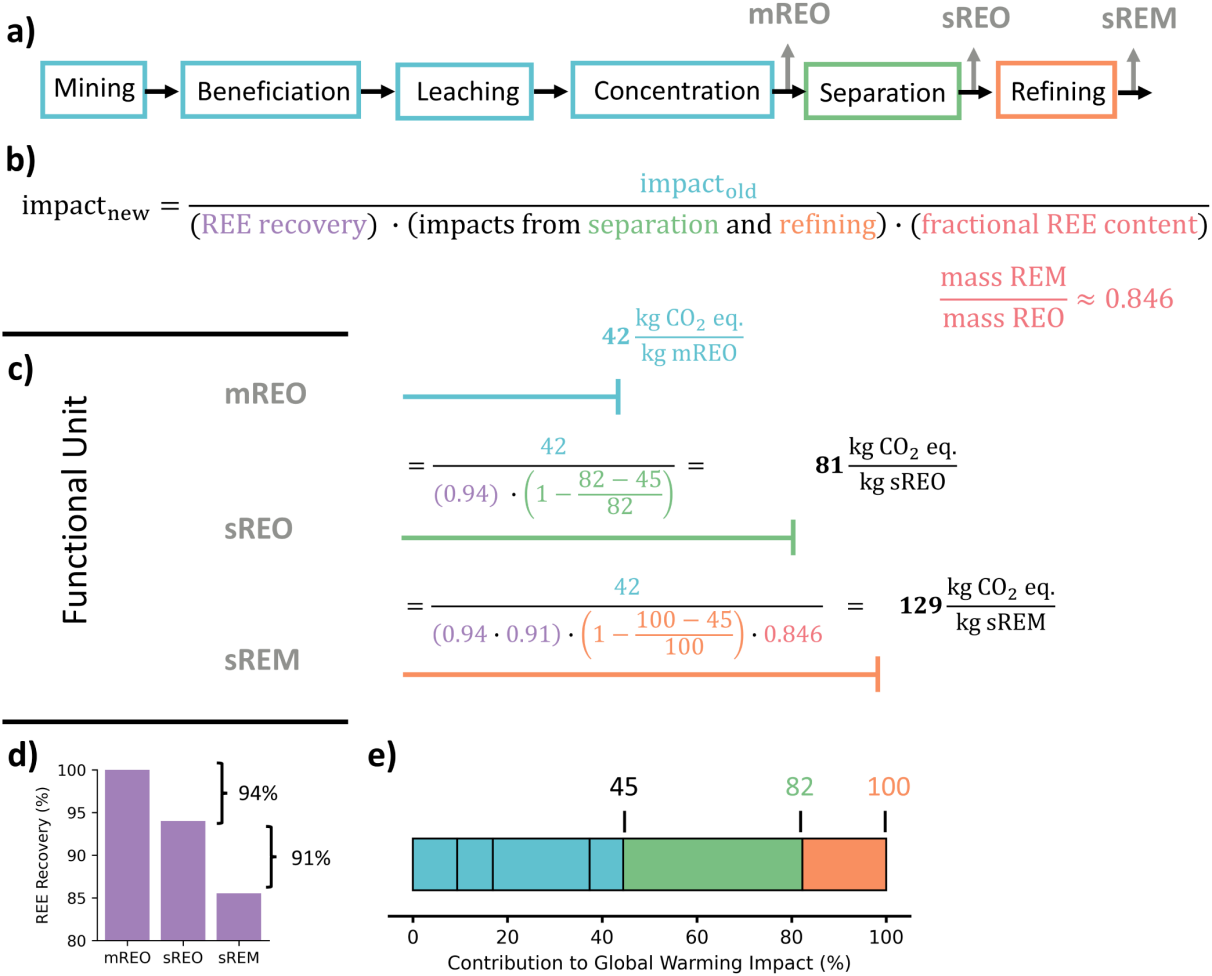
One example of how to convert between functional units used in REE production studies. This example considers that a study (with a global warming impact of 42 kg CO_2_eq/kg mREO) wants to estimate the total global warming impact after separation and refining of that mREO product into sREO and sREM. Panel a shows a flowsheet to produce the different REE products broken down by process sections. Panel b shows the generic equation for converting between functional units with each term color coded for clarity. Panel c shows the three functional units that can be converted between (mREO, sREO, and sREM). Panel d shows the average amount of REEs recovered after additional processing where 100% of REEs are initially present as the mREO product. Panel e shows, on average, how much each process section contributes to the total impact of producing REMs for the Bayan Obo processing route.

The functional unit conversion in Figure 6 is an illustrative example and is not intended to define a single universal conversion route for all REE systems. We do not suggest harmonizing across different feedstocks (e.g., monazite vs bastnaesite), since each route requires a distinct process chain with unique emissions and coproduct profiles. Rather, this conversion provides a simplified framework that can be adapted using the most relevant example system and process section data available. We recommend that analysts select conversion parameters from the processing route most similar to their system of interest, recognizing that differences in ore composition, system scope, and recovery efficiency will influence results. Therefore, uncertainty related to these parameters should be quantified when using this method. With careful analysis, this method can aid comparability across studies while acknowledging the inherent limitations of data availability and REE system diversity.

#### 3.2.2 Multifunctionality and Allocation

ISO standards^11,12^ describe how systems with multiple functions or products distribute, or allocate, environmental impacts among various outputs of a system. In the context of REE recovery, both conventional and unconventional feedstocks produce coproducts in addition to REEs (e.g., iron ore at Bayan Obo). Therefore, it is necessary to decide how to allocate process burdens to these coproducts. For iron (Fe) and niobium (Nb) ore coproduced at BO, a two-step economic allocation has been performed to attribute some of the impacts to these products.^52,79^ The influence of this decision has not been rigorously explored. In other cases, when the coproduct is highly valuable (e.g., electronics with trace REO, copper (Cu), and gold (Au)), the impacts from REEs can range orders of magnitude (or even become negative) depending on the allocation procedure used. Therefore, it is important to carefully consider how to allocate impacts to both coproducts and among individual REEs. Below, we step through the ISO standards and summarize the different allocation approaches used in the LCAs of REE systems and their limitations.

For certain functional units, such as 1 kg of mREE produced or 1 kg sREO produced, allocation between individual REEs is not required. This approach is simple and convenient, making comparisons between different systems easy at first glance. However, different feedstocks and processing technologies lead to differing compositions of REE products. For example, ion adsorption clays have a higher composition of heavy REEs than monazite and bastnaesite. Heavy REEs are typically less abundant and have higher value compared to light REEs. This discrepancy makes direct apples-to-apples comparisons impossible, as all forms of “1 kg of separated REOs” are not equivalent in their value and end use.

The ISO 14044 standard^12^ first recommends avoiding allocation through system expansion and system subdivision. During separation, all REEs are dependent on the initial separation step that produces the first individual REE, but only the remaining REEs are dependent on further downstream separations. Therefore, subdivision has been used in some studies to reflect this partial independence.^39,48^ However, subdivision is questionable due to the physical (e.g., shared solvent) and economic dependence of the separation on each REE.^18,80^ In the literature reviewed, it was commonly unclear how the subdivision had been performed making it difficult to compare between studies. System expansion may also be inappropriate as the “displaced” processes may not exist in reality.^79^

Continuing along the ISO 14044 guidelines,^12^ impacts can be attributed to individual products by allocating based on physical relationships (e.g., mass, energy), or, if necessary, economic value. Within the REO literature reviewed, numerous allocation methods have been used to attribute impacts to iREOs.^39,40,81^ However, the most commonly used allocation method is economic allocation, where the mass and price of each coproduct is used to determine how to distribute the impact. Several studies have shown how mass allocation can shift impacts to the more abundant light REEs while economic allocation shifts impact to heavy REEs, which are typically of higher value.^40,49^ Therefore, if a functional unit of 1 kg of lanthanum (La) oxide produced (a light REE) is used and economic allocation is performed, the system would have very low impact since most of the impact has been attributed to the heavy REE products. Many studies in the field do well to emphasize these limitations. However, a more involved discussion reporting multiple functional units and allocation methods is frequently not examined, such as a sensitivity analysis as outlined by ISO standards. Further, economic allocation relies on highly variable and uncertain REO prices, as described in Section 3.1.2. This variability will likely extend into the future as REO demand is projected to increase (e.g., to supply the clean energy transition)^2^ and new feedstocks with different compositions of REOs are utilized.^3^ Therefore, it is important that studies using allocation are fully transparent with their allocation factors to allow for modifications as the value of individual REOs change over time.

Replication of studies using subdivision or other allocation methods is often difficult or impossible without detailed methodological descriptions and transparent data. Even for the widely utilized ecoinvent database, it was challenging to convert the reported impacts (per kg of iREO) to per kg of sREO. Using ecoinvent’s methods, a mass-based subdivision is performed for reference products followed by economic allocation to both reference products and allocatable byproducts. Though this method is common practice within ecoinvent, the methodology is not easily accessible nor is the ecoQuery documentation^82^ sufficiently descriptive to replicate these results. EcoQuery does make clear whether REOs are reference products or allocatable byproducts, or explain the basis for their classification, which does not appear to rely on mass or market value.^82^ Since allocated impacts for REEs depends on these designations, the impact results may be ambiguous, lacking consistency or methodological rigor. Further, since reverting this allocation is challenging, it is difficult to quantify this uncertainty and to compare between different functional units. Overall, the problems identified in the ecoinvent data set are just one example of how multifunctionality is reducing the reliability of LCA results and their utility in assessing novel REE production systems.

#### 3.2.3 Limited Data Availability for Life Cycle Inventories (LCI)

Many of the challenges associated with LCI collection for conventional systems are covered in two recent reviews.^18,19^ For one, primary data availability (from the actual processes) for conventional REO production routes is sparse with few studies having direct measurements from industry. Therefore, many studies have used assumptions and estimations to fill data gaps (e.g., using regulatory information for toxic waste management). Some mistakes have been found in these calculations and in the implementation of assumptions (e.g., REE content of ores).^19^ Many studies have used parts of previous works in their LCIs leading to few truly distinct LCIs. The use of similar foundational assumptions could artificially decrease the variation in results, leading to a false sense of accuracy. Over time, studies have begun to fill these gaps (e.g., solvent extraction), but primary data is still needed. Further, there is significant illegal production of REOs, where even less is known about these systems (as much as 40% of overall production from IAC in China).^37^ Illegal mining impacts were estimated to be around 215% of the impact of conventional mining.^37^ In addition to limited site-specific data, background LCI data has been missing for some flows. A few of these chemicals include P_2_O_4_ (used in solvent extraction)^79^ and oxalic acid (used in REE precipitation).^40,54^ In more recent studies we reviewed with updated LCI information and sensitivity analysis, the impact of these chemicals has proven significant.^44,46^ Overall, despite over 15 LCAs for conventional REO production, there is large uncertainty in the LCAs of conventional systems that has not been quantified by rigorous uncertainty analysis.

For novel systems from secondary sources, many of these REE extraction and processing technologies are still in early stages of development, where full-scale primary data does not yet exist. Therefore, performing LCAs on emerging technologies has several challenges including comparability, scale, data, and uncertainty.^32^ Issues with comparability relate to system boundary and functional unit, as discussed in Section 3.2.1. Additionally, these novel systems primarily rely on lab-scale data, which likely overestimates impacts compared to optimized, full-scale systems. The data required to model these steps accurately may be sparse or inconsistent due to the technical complexity of REE extraction and separation (e.g., bioleaching or sorption) from unconventional sources. As these new technologies develop, background databases may not have the chemicals needed for these new or specific technologies requiring the use of proxy chemicals. Therefore, these novel systems may have large uncertainty making it challenging to compare to conventional systems and to identify future direction for technology development.^32^

#### 3.2.4 Limitations of the Current Impact Assessment Methodology

Life cycle impact assessment (LCIA) methods are used to quantify the impact of flows to the environment for specific impact categories. For a minority of impact categories (e.g., global warming, ozone depletion), the methods to quantify environmental impacts are largely consistent due to standardized frameworks established by the International Panel on Climate Change (IPCC)^83^ and World Meteorological Organization (WMO).^84^ For the other impact categories, impacts from different methodologies can vary substantially, even if they employ the same units, due to different assumptions that influence characterization factors. Therefore, it is difficult to compare between studies that use different LCIA methods. In the REE literature reviewed, many LCIA methods have been used including CML, ILCD, TRACI, and ReCiPe. Further, within each LCIA method, there have been different versions which have their own impact categories and characterization factors.

When choosing an LCIA methodology to use, it is essential to consider all relevant impact categories to understand potential trade-offs. While regulatory development in the United States is still in progress, under the European Union regulatory context on LCA, the Commission Recommendation 2021/2279 ^85^ requires that “all EF impact categories shall be applied, without exclusion.” Following that, the EF framework mandates the comprehensive application of impact categories, ensuring methodological consistency across Product Environmental Footprint studies.

In the context of critical mineral and rare earth element recovery, regulatory frameworks in the U.S. are still under development. In the European Union, in 2024, the Critical Raw Materials (CRM) Act^86^ introduces targeted environmental footprint reporting for critical raw materials. Under Article 31 of the CRM Act, Environmental Footprint Declaration, it is required that “verification of the environmental footprint of different critical raw materials shall consider scientifically sound assessment methods and relevant international standards.” The calculation and verification rules must identify *at least three most relevant environmental impact categories* that account for the majority of the overall environmental footprint, one of which must be greenhouse gas emissions. For REO production, some impact categories are frequently reported (e.g., global warming, acidification, ecotoxicity). However, some other relevant, but less developed, impact categories are irregularly examined. Here, we discuss a few of the “relevant environmental impact categories” in rare earth element recovery LCAs, including radioactivity, water use, and long-term impacts of waste storage, and address the limitations of current LCIA methodology in quantifying impacts in these areas.

##### 3.2.4.1 Impacts from Radioactivity

During the processing of ores and industrial wastes containing REEs, commonly radionuclides (e.g., Uranium, U and Thorium, Th) entire the ecosphere from fine particle and fluid releases. However, quantifying the impacts of these radionuclides has two main challenges. One challenge is the lack of data on the amount and concentration of radionuclides crossing the system boundary. For conventional REE systems, radioactive wastes are produced during beneficiation, leaching, and extraction when the radionuclides are extracted from the feedstock alongside the REEs. The end fate of these metals is generally long-term, acid mine waste storage sites. These waste sites have varying levels of management where there is the possibility of toxic metals leaching into the environment. Some notable examples of the human impacts of this waste management are examined in Section 3.5. Beyond quantifying the radioactive flows, it is difficult for LCIA methods to determine the impact of these flows. Some LCIA methods (e.g., TRACI v2.1)^87^ do not contain common flows from mining like uranium (U) and thorium (Th). Other LCIA methods (e.g., ReCiPe 2016)^88^ consider radioactive flows. However, the methodology used to quantify the impact of these radioactive flows is still developing. First, radionuclide releases are commonly irregular discharges (e.g., break in containment), which is typically not considered when conducting LCAs. Further, after these releases, it is difficult to model how radionuclides move and accumulate in the environment, which can be dependent on geography. Beyond challenges modeling the movement of radionuclides through the environment, directly relating radiation exposure to human health impact is difficult since long-term low-dose radiation can express as diseases now and in future generations.^89^ Further discussion of the implications of radionuclide impacts for REE systems is discussed in other reviews.^18,19^ Ultimately, radiological impacts are likely undercounted due to inventory gaps and evolving characterization. Studies should clearly state assumptions related to radionuclide flows and report sensitivity to scope and method choice. Additionally, under ISO Standard 14042/44, an LCA is designed to evaluate the potential environmental aspects and impacts of a product system throughout its life cycle under *normal operating conditions*, therefore, for the evaluation of REE systems, a separate risk assessment is encouraged to analyze the risk of accidental release.^90^

##### 3.2.4.2 Water Use and Depletion Impacts

There are large amounts of water used in REE production systems during processes like leaching, mining, and solvent extraction. However, there are inconsistencies in how water use impacts are modeled across LCIA methods.^77^ One key detail is defining water use versus water depletion. Water use quantifies the amount of water used by a system, while water depletion is the amount of water consumed by the system that will not return to its original aquifer. When considering water depletion, a system that recovers REEs from wastewater while also treating that wastewater would receive a credit for returning water to the environment. However, in common LCIA methods, such asReCiPe 2016 (v1.1), it is unclear whether this treated wastewater is considered a credit within the system boundary. In the ReCiPe report,^91^ due to the complexity of linking water consumption to biosphere flows, implementation is left to interpretation. We examined implementations of ReCiPe water depletion from both openLCA and ecoinvent (v3.9.1). The openLCA implementation includes water flows back to the environment as a credit (characterization factor of −1), while ecoinvent’s implementation does not. In wastewater treatment literature, it is acknowledged that most existing LCIA methods do not account for water flows back to the environment, which is important for water reclamation systems.^92^ Their recommendation is to use the LCIA method called AWARE (Available WAter REmaining),^93^ which links flows to water depletion impact. However, this LCIA method is not implemented in ecoinvent making it challenging to perform the analysis in some programs. Understanding the fate of water in REE production systems (e.g., indefinite storage as acidic tailings) is critical for managing the water, food, and energy nexus, especially in geographies with water scarcity.

##### 3.2.4.3 Impacts of Long-Term Waste Storage

In the United States, 20% of the mining facilities inspected by the environmental protection agency (EPA) between 1990 and 1995 violated regulations from the Clean Air Act, Clean Water Act, and Resource Conservation and Recovery Act (RCRA) due to mismanagement of acid drainage and tailings disposal.^94^ Further, secondary REE feedstocks (e.g., coal fly ash) and wastes from REE production are classified as Technologically Enhanced Naturally Occurring Radioactive Materials, which are regulated under the Clean Air Act, National Emission Standards for Hazardous Air Pollutants, and more recently the RCRA as a mixed waste.^95,96^ Therefore, the environmental impact of these wastes must be quantified in LCAs of REE production systems from both primary and secondary feedstocks. However, there are currently no standardized methods, and it is unclear how to best quantify the impacts of these wastes in LCA. Do these wastes actively leak into the environment? Should studies consider sporadic impacts from large accidental releases of toxic waste? What is the end fate of waste if the management structure collapses after a century of active management? How questions like these are answered may lead to impacts ranging up to 8 orders of magnitude for landfill metal emissions.^97^ To date, there is no consensus on whether long-term emissions should be considered in LCA. A report from ecoinvent gives a detailed discussion of the arguments for, and against, including long-term emissions in LCA. Further, the ecoinvent database includes two versions of each impact method (“w/LT” including long-term emissions and “w/o LT” ignoring the emissions) to help address this issue.^98^ Other studies have suggested the integration of risk assessment, which quantifies the probability and consequence of accidental releases, with conventional LCA. Though careful hybridization of these approaches is encouraged,^99,100^ there is no consensus on how to conduct this integrated analysis. Although it is unclear whether, and how, long-term emissions should be quantified, these decisions can greatly influence conclusions about the sustainability of a system (e.g., for waste remediation and accumulation).

We advocate for application of established standardized methods and continued methodological development efforts in these areas. In the meantime, since radioactivity, water depletion, and long-term waste are inconsistently characterized across LCIA methods, authors should state method choices (e.g., AWARE for water), state time-horizon assumptions, and test their influence on conclusions using scenario and sensitivity analyses.

### 3.3 Uncertainty, Sensitivity, and Scenario Analysis

Sensitivity analysis quantifies the relationship between input uncertainty and model outputs (e.g., sustainability metrics like NPV and GW impact), which is essential for establishing defensible conclusions from a model. In the LCA and TEA studies we reviewed, primarily, local sensitivity analysis was performed, where parameters were individually varied (typically ±20%) to quantify how each parameter affected sustainability metrics. However, these local sensitivity analyses did not define realistic ranges of input uncertainty nor consider the possibility of interactions between parameters. Global sensitivity methods can vary multiple parameters simultaneously from well-defined uncertainty distributions (e.g., Monte Carlo),^101–103^ but these global methods were rarely performed in the reviewed studies (4 of 17 TEA studies). Instead, most studies performed scenario analyses that determined the range of possible outcomes using best- and worst-case scenarios of parameters (e.g., technological performance and market prices). Future works must go beyond varying individual parameters to define a range of outcomes. Using global methods, we can more rigorously quantify the likelihood of achieving a specific sustainability goal under uncertainty. This probabilistic understanding of system sustainability can be used to more clearly identify research targets for influential parameters, help decision makers understand uncertainties in the model, and identify research direction in large decision spaces.

### 3.4 Disconnection between Economic and Environmental Assessments

By understanding the trade-offs between profitability, environmental, and social impact categories over the product lifecycle, decisions can be made early in technology development to optimize system sustainability. In many REE recovery studies, LCA and TEA are conducted separately, often using different assumptions, system boundaries, and contextual values. This disconnection can lead to inconsistencies and even misleading conclusions. For instance, most of the LCA studies of conventional REE production consider the selective intra-REE separation and refining steps within the scope of the system (Table 2). However, 7 of the 17 TEA studies did not include these process sections. Therefore, very different REE product values were used to determine profitability in TEA and economic allocation in the LCA studies. Further, many of the systems reviewed led to the generation or remediation of waste. The LCA studies made assumptions about how strictly the waste management would be held to regulations, while TEA studies assigned costs to treat or manage these wastes that do not always align with the LCA assumptions (e.g., assume costs or impacts of waste management are negligible). For example, one study determined that most of the revenue of REE production was from the remediation of the waste they used as a feedstock.^27^ These are just two examples of how the lack of integration can lead to an incomplete understanding of the overall sustainability of a technology or process.

Such misalignments highlight the need for integrated sustainability assessment, where model inputs to environmental and economic evaluations are harmonized. Aligning assumptions, data sources, and methodological choices across LCA and TEA ensures a more accurate and holistic understanding of trade-offs and synergies, ultimately supporting better-informed decision-making in research, development, and policy.

### 3.5 Emerging Social Impact Assessments

Social life cycle assessment (sLCA) enables early identification of potential social risks and stakeholder concerns. This is particularly critical in the context of REE recovery, as many REEs are traditionally sourced from regions with poor labor practices, environmental justice issues, and other societal challenges. Assessing social sustainability in emerging REE recovery pathways will help to reduce reliance on exploitative supply chains, promote fair labor practices, and support a more equitable resource distribution. Despite its importance, the least considered pillar of sustainability is social impact. Of the critical minerals, REEs are among the least explored in the social sciences despite many stories of sociological harm to communities.^9^ Near Baotou in China, people have cited livestock and crops dying after the start of REE mining in the region, resulting in a decrease in one town’s population from 2,000 to 300 people within 10 years.^21,104,105^ The Mountain Pass mine in the U.S. had 60 wastewater spills amounting to 600,000 gallons of toxic wastewater entering the environment. Mismanagement here likely led to the opposition of a new REE mine in Minnesota, where 98% of comments from the public were negative.^21,104^ In Malaysia, a past radioactive materials leak led to lawsuits claiming environmental damages that were ultimately refuted by the courts. Consequently, when a new REE refining site was permitted in Kuantan, locals had environmental concerns.^104^

These stories highlight some of the sociological issues pertaining to REE production globally. Broadly, in the literature, socio-ecological concerns revolve around three connected issues: engagement of local communities, environmental justice, and the cost of mitigating impacts.^9,21,104–106^ First, indigenous and local communities near REE resources are directly affected by the impacts of REE production and, therefore, should be engaged in the permitting process. However, in many cases in critical mineral production, companies obtain permission from state governments without communicating with the local community. As each culture is unique, failing to engage these local communities has led to loss of livelihood (e.g., farmers), dispossession of land, civil unrest, and, in extreme cases, war.^105^ Greater communication with local communities can ensure that wealth generation extends to improving opportunities for these communities. In addition, those affected tend to be from disadvantaged communities, particularly in the Global South where environmental and human health regulations are weaker.^105,106^ Strict regulations are commonly framed as making operations less economically competitive. Therefore, it is critical to quantify these trade-offs between profit, environmental, and social impact to all stakeholders. The conversation about the cost of cleaning up hazardous waste is primarily confined to after the damage has occurred. However, the multigenerational diseases that occur from exposure to these wastes can shift the cost from businesses to individuals and social systems. Therefore, we should prioritize limiting impacts from before and during operation since this period has a limited time horizon and is geographically contained to the site of production thereby decreasing the total cost to businesses and society.^106^ However, robust methodologies that quantify potential impacts prior to production are required.

Of the three pillars of sustainability, social sustainability analysis methods are the newest and least developed. There has been significant progress in developing and supporting social impact assessment resulting in the United Nations Environment Programme–Society of Environmental Toxicology and Chemistry (UNEP-SETAC) Life Cycle Initiative (sLCA) Framework that is distributed in two documents^16,17^ and an ISO standard.^107^ The sLCA methodology is similar to environmental LCA in that it follows ISO 14040/14044 standards and has familiar phases for the analysis (goal and scope definition, LCI, LCIA, interpretation). The sLCA methodology has primarily been applied to waste management and biobased product systems.^108^ However, there have been a number of studies relating to critical mineral and REE mining.^109–115^ Of these studies, only two relate specifically to REO production, though they both originate from the same group of researchers.^112,113^ They found that corruption, bribery, and fair competition in REO and magnet production supply chains pose risks to social structures. Both studies found the Mountain Pass and Mount Weld mines have the least social impact while the Chinese and Malaysian systems have the highest impact. However, these studies are limited in several ways. For example, these studies did not perform interviews with local communities to supplement their data nor use actual price data as inputs with the PSILCA database. In addition, missing data in China and Malaysia processes is treated as “low risk”, which could lead to an underestimation of impacts for the Bayan Obo and Mount Weld supply chains. Further, since the PSILCA database only supports the activity variable of “worker hours”, the relevance of the results is limited to the “worker” stakeholder group.^112,113^

Ultimately, sLCA methods require further development. Specifically, sLCA methodologies must have better standardization to enable comparisons between studies. Data availability and quality need to improve, especially for local site-specific data.^109^ To this end, some background databases exist (PSILCA and SHDB) and can be used in common LCA software like openLCA and SimaPro. However, database scopes have limited data and stakeholder groups available, leading to results that may not accurately quantify all social impacts of a system. Further, there is no direct relationship between sLCA impact categories and the UN sustainable development goals leading to incomplete coverage of discrimination issues.^108^ Despite these limitations, sLCA has been helpful in identifying potential hotspots in prospective system designs. Highlighting these hotspots as part of the discussion around novel REO production systems can help increase awareness of common social impact vulnerabilities and encourage smarter designs in the future.^21,111^

## 4 Recommendations and Conclusions

Many of the methodological variations highlighted in this review (e.g., LCIA method selection, allocation choices, and sLCA stakeholder coverage) are not unique to REE systems, and there will not be a single solution tailored to REEs alone. Nevertheless, the unique features of REE production—such as coproduct allocation, ore composition diversity, and location-specific regulatory and social contexts—amplify the influence of these broader issues necessitating more standardized methods. From this review, we recommend the following to advance this field:

### 4.1 Standardize and Integrate Methodologies for Evaluating REE Recovery Systems

In TEA studies, profitability is largely tied to revenue from REEs, which is highly variable. We recommend reporting profitability by the indicator minimum selling price (MSP), as its calculation does not rely on the price of the main product. Therefore, TEA studies will remain relevant regardless of REE price fluctuations allowing direct comparison with future studies. Additionally, low TRL systems can be expected to become more optimized as the technology develops resulting in improved profitability. Not considering this development could disincentivize emerging technologies. The profitability of an *N*th-of-a-kind plant can be estimated based on the cost of the first-of-a-kind plant and a learning rate based on different methodologies (e.g., from NETL, EIA).^116^ Some other LCA/TEA methods (e.g., Lang factors, product value identification, coproduct handling, and DCF analysis, etc.) as well as characterization factors applied in REE and critical mineral recovery studies, need to be further studied and standardized by researchers in this field to improve consistency and methodological rigor.

In LCA studies, we recommend all systems report a common mREE or sREE functional unit and the REE composition for enhanced comparability without uncertainties due to allocation. Because REEs are globally traded commodities, cross-system comparisons are both meaningful and necessary for evaluating sustainability across production routes. At the same time, we recognize that location-specific requirements (e.g., environmental regulations, social context), ore compositions, and product specifications inherently lead to differences in results. To account for this, we further recommend that systems with unique features report additional functional units with careful descriptions of their allocation procedures (e.g., iREO, Nd for magnets) to contextualize results for these specific applications. Further, harmonization of studies within each processing route should be completed with more rigorous uncertainty and sensitivity analysis, as outlined in point 3 below. Altogether, harmonized assumptions and inventories across LCA and TEA, combined with more standardized terminology and methodology, will help evaluate different systems and REE recovery processes consistently, enable informed comparisons among emerging technologies, and allow trade-offs to be assessed systematically across different dimensions of sustainability and feasibility.

### 4.2 Enable Comprehensive Assessment and Understanding of Impacts from Radioactivity, Water Use, and Waste Handling and Treatment

Although radioactivity, water use, and waste management are regulated by governments and significantly influence the profitability of REE production systems, they are infrequently included within the scope of literature studies. This exclusion reflects the limited inventory and cost data, as well as the absence of established standards or best practices for quantifying waste management costs and environmental impacts of REE systems. We recommend that future profitability assessments include both capital and operational expenses related to waste management. To quantify environmental impacts using current LCIA methods, we suggest applying ReCiPe 2016 for radioactive substances and AWARE for water depletion. However, further methodological development and improved completeness are needed to enable more robust impact assessments related to radioactivity, water use, and waste. Improving data availability and advancing LCIA methodologies will be essential for developing a comprehensive understanding of the costs and environmental impacts associated with REE production.

### 4.3 Account for Uncertainties and Expand Scenario Analysis

Global scenario, uncertainty, and sensitivity analysis methods that simultaneously consider uncertainties from parameters (e.g., reactor temperature or electricity price) and scenarios (e.g., functional unit of iREO vs sREO) are essential for comprehensive sustainability assessments. Comparing one number to another ignores the complexity of these systems and leads to erroneous conclusions (e.g., GW impact of ethanol fuels).^117^ Stochastic methods, like Monte Carlo analysis, have been used in other fields and are equipped to evaluate the entire design space. As opposed to varying parameters by ±20%, physically reasonable limits should be used to bound parameters and understand indicator sensitivity. Targets for key parameters can be identified based on different profitability and environmental goals to more clearly guide future research efforts.^118^ In addition, methodological studies should explore how including model uncertainty (e.g., LCIA methods, long-term impacts) may influence conclusions on the sustainability of these REE systems to avoid pursuing unsustainable paths.^119^

### 4.4 Incorporate Social Sustainability into the REE Field

Social impacts remain underexplored but are critical in REE supply chains. Including social life cycle assessment (sLCA) enables early identification of risks and supports fair, responsible sourcing of REEs. Ideally, TEA, LCA, and sLCA would be completed together for greatest value. However, sLCA is still a developing methodology. Therefore, we recommend that TEA and LCA studies discuss the potential social risk factors associated with their systems qualitatively. With good data transparency, future sLCA studies can leverage these data to quantify the social impacts and hotspots of emerging systems.

In summary, this review has identified key challenges in the sustainability assessments of REE production systems and highlighted the needs to standardize the approaches for enabling comparisons between systems. Further, as more data and studies become available in this field, we expect that solutions to the above-mentioned challenges will become clearer. These system-level sustainability analyses will be instrumental in ensuring the supply of REEs for consumer and clean energy technologies in the present, while supporting long-term resource availability and sustainability for future generations.

## Supplementary Material



## Notes

Additional discussion of methods; full tables of data used in the main text (PDF)

## Abbreviations

**Acronym**: **Definition**
AMD: Acid Mine Drainage
BO: Bayan Obo (China)
CAPEX: Capital Expenditure
CEPCI: Chemical Engineering Plant Cost Index
CRM: Critical Raw Materials
DCF: Discounted Cash Flow
DOE: Department of Energy
EPA: Environmental Protection Agency
FCC: Fluid Catalytic Cracking
FLP: Fluorescent Lamp Powder
GW: Global Warming
ICIS: Independent Commodity Intelligence Services
ILCD: International Reference Life Cycle Data System
IPCC: Intergovernmental Panel on Climate Change
ISO: International Organization for Standardization
LCA: Life Cycle Assessment
LCI: Life Cycle Inventory
LCIA: Life Cycle Impact Assessment
MP: Mountain Pass (United States)
MSP: Minimum Selling Price
MW: Mount Weld (Australia)
NiMH: Nickel–Metal Hydride (a battery)
NPV: Net Present Value
OPEX: Operational Expenditure
PSILCA: Product Social Impact Life Cycle Assessment Database
sLCA: Social Life Cycle Assessment
SP: Southern Provinces (China)
TEA: Techno-Economic Analysis
TRL: Technology Readiness Level
UNEP-SETAC: United Nations Environment Programme–Society of Environmental Toxicology and Chemistry
WMO: World Meteorological Organization

## Types of Rare Earth Products

mREE: Mixed Rare Earth Element (mixture of several rare earth elements)
REE: Rare Earth Element
REEp: Highly Processed Rare Earth Product (e.g., NdFeB magnet)
REO: Rare Earth Oxide
REM: Rare Earth Metal
iREE: Individual Rare Earth Element (pure salable individual rare earth element)
iREO: Individual Rare Earth Oxide (pure salable individual rare earth oxide)
iREM: Individual Rare Earth Metal (pure salable individual rare earth metal)
sREE: Separated Rare Earth Elements (sum of individual rare earth elements)
sREO: Separated Rare Earth Oxide (sum of individual rare earth oxides)
sREM: Separated Rare Earth Metal (sum of individual rare earth metals)

## Life Cycle Impact Assessment Methods

AWARE: Available WAter REmaining (water scarcity footprint model)
CED: Cumulative Energy Demand
CML: Centrum voor Milieukunde Leiden (Leiden University LCA method)
Eco-costs: LCIA approach quantifying external costs of environmental burdens
EF: Environmental Footprint (EU LCIA method)
EI99/EI95: Eco-Indicator 99/95 (LCIA methods)
ReCiPe: Rijksinstituut voor Volksgezondheid en Milieu Combined Indicators for Environmental Impact Assessment
TRACI: Tool for the Reduction and Assessment of Chemicals and Other Environmental Impacts
USEtox: United Nations Environmental Programme model for human/ecotoxicity
