# Supplementary material for: Toward Sustainable Rare Earth Element Production: Key Challenges in Techno-Economic, Life Cycle, and Social Impact Assessment
Source: ACS Sustain Chem Eng. 2026 Feb 3;14(6):2733–51. doi: 10.1021/acssuschemeng.5c06147 (PMC12914704; doi:10.1021/acssuschemeng.5c06147)
Supplement: Supplementary file 1 [file sc5c06147_si_001.pdf]

Toward Sustainable Rare Earth Element Production: Key Challenges in Techno-Economic, Life Cycle, and Social Impact Assessment

Adam Smerigan[1,2] and Rui Shi[1,3]*

[1]Department of Chemical Engineering, The Pennsylvania State University, University Park, Pennsylvania 16802, United States

[2]Lawrence Livermore National Lab, Livermore, CA 94550, United States

[3]Institute of Energy and the Environment, the Pennsylvania State University, University Park, Pennsylvania 16802, United States

*Corresponding author: rms6987@psu.edu

Contents

1 Calculation of the Adjustment Factor for Illegal Mining of REEs...S2

2 Compiled LCA Data S3

3 Compiled TEA data...S5

4 Functional Unit Conversion Method...S8

5 Equations for calculating profitability S9

6 References S10

1 Calculation of the Adjustment Factor for Illegal Mining of REEs

Since impacts from illegal REE mining are significant, we suggest that it should be considered when comparing to the environmental impacts of developing systems in other regions. To estimate the additional impacts of illegal REE production in China, we calculated an "adjustment factor". This adjustment factor used literature data on the quantified impacts of illegal REE mining and the capacity of illegal mining to estimate how much additional impact could be attributed to Chinese REE production. For ease of use, we averaged this adjustment factor across all impact categories and production routes. However, a more accurate analysis could be done using this method for individual impact categories and production routes. To use this adjustment factor, any environmental impact from conventional REE mining in Chinese production routes can be multiplied by the adjustment factor to estimate the additional impact from illegal mining in these regions.

Table S1: Ratio of environmental impacts from illegal mining to legal mining for three production routes. Original data from Lee and Wen 2018.[1]

	Production Route			
Impact Category	Bayan Obo	Sichuan	Southern Provinces	Average
Acidification	5.88	1.34	1.89	
Ecotoxicity	1.57	1.60	1.54	
Eutrophication	5.72	4.63	2.54	
Fine Dust	2.10	1.35	1.80	
Human Toxicity	1.68	1.41	1.51	
Global Warming	1.89	1.37	1.71	
Photochemical Oxidation	2.23	1.18	1.47	
Chinese Abiotic Depletion Potential	2.01	1.42	1.66	
Average across categories	2.89	1.79	1.76	2.15
Standard Deviation	1.81	1.15	0.35	

Table S2: Calculation of the factor used to adjust the impacts of legal REE mining in China considering the additional impacts of illegal mining at these locations.

Parameter	Value
Percentage of REOs that are produced illegally (average of scenarios from Lee and Wen - 2018)	17
Ratio of illegal to legal mining impacts (averaged across production routes)	2.15
Impact from illegal mining	0.17 * 2.15 = 0.359
Impacts of legal mining	1 - 0.17 = 0.83
Adjustment factor for illegal REE mining	0.359 + 0.83 = 1.19

2 Compiled LCA Data

Table S3: Summary of LCA studies reviewed. Locations for conventional REE production are Bayan Obo (BO), China Southern Provinces (SP), Mount Weld (MW), and Mountain Pass (MP).

Author Name	Reference	Date	Feedstock	Paper Focus	Location	Functional Unit
Arshi	2	2018	Conventional	Analysis	BO, SP	iREO, iREM, REEp
Bailey	3	2020	Conventional	Review	BO, SP, MW	sREO
Marx	4	2018	Conventional	Analysis	BO, MW, MP	REEp
Zaimes	5	2015	Conventional	Analysis	BO	mREO
Deng	6	2019	Conventional	Analysis	SP	mREO
Lee	7	2017	Conventional	Analysis	BO, SP	iREO, sREO
Lee	8	2018	Conventional	Harmonization	BO, SP, SC	sREM
Zapp	9	2018	Conventional	Analysis	BO, SP, NK	iREO, sREO
Zapp	10	2022	Conventional	Analysis	all	iREO
Vahidi	11	2016	Conventional	Analysis	SP	mREO
Vahidi	12	2017	Conventional	Separation	BO, SP	mREE
Vahidi	13	2018	Conventional	Refining	SP	iREF, iREM
Schreiber	14	2016	Conventional	Analysis	BO, NK	iREM
Schreiber	15	2021	Conventional	Review	BO, SP	iREO, sREO
Koltun	16	2014	Conventional	Analysis	BO	iREO
Koltun	17	2020	Conventional	Analysis	MW	iREO, sREO
Schulze	18	2017	Conventional	Analysis	SP	sREO
Sprecher	19	2014	Both	Analysis	BO	REEp, sREO
Brown	20	2024	Secondary	Analysis	US	Soil processed
Ippolito	21	2021	Secondary	Analysis	Italy	Fluorescent powder processed
Kulczycka	22	2016	Secondary	Analysis	Poland	PG treated
Alipanah	23	2020	Secondary	Analysis	US	mREO
Rabbani	24	2024	Secondary	Analysis	US	mREOH, sREO
Chowdhury	25	2021	Secondary	Analysis	US	mREO
Jin	26	2016	Secondary	Analysis	-	REEp
Li	27	2019	Secondary	Analysis	US	Gold
Sanchez Moran	28	2024	Secondary	Analysis	US	mREO
Van Nielen	29	2024	Secondary	Analysis	Europe	REEp
Liu	30	2020	Secondary	Analysis	US	mREO, lighting service
Thompson	31	2018	Secondary	Analysis	US	FCC waste processed
Magrini	32	2022	Secondary	Analysis	Sweden	mREO

Table S4: Further information about the LCA studies reviewed. If mREEs are separated into sREO products, an "X" is marked in the "separate" column. If sREOs are refined into sREM products, an "X" is marked in the "refine" column.

Author Name	Reference	Coproduct	Allocation	separation	refining	Hot Spot Analysis	LCI database
Arshi	2	Iron ore, REOs	E	X	X	X	EI, other
Bailey	3	Iron ore	E, S	X		X	EI, Thinkstep
Marx	4	Iron ore, REOs	E, M	X	X	X	EI 3.3
Zaimes	5	Iron ore, REOs	E, M, O				EI 3
Deng	6	-	-			X	EI 3
Lee	7	Iron ore, REOs	E, M	X	X	X	CLCD
Lee	8	Iron ore	-	X	X		CLCD
Zapp	9	Iron ore, REOs	E, S	X	X	X	EI 3.3
Zapp	10	Iron ore, REOs	n.a.	X	X	X	n.a.
Vahidi	11	-	E				EI 3.0
Vahidi	12	REOs	E	X		X	EI 3.0
Vahidi	13	REOs	E	X	X	X	EI 3.0
Schreiber	14	Iron ore, REOs	E, S	X	X	X	GaBi6, EI 2.2
Schreiber	15	-	-	X			-
Koltun	16	Iron ore, REOs	E, M	X		X	EI
Koltun	17	REOs	E	X			EI
Schulze	18	-	-	X			EI 3.2
Sprecher	19	Iron ore, REOs	E	X	X		EI 2.2
Brown	20	-	-				AGRI-BALYSE
Ippolito	21	mREO	D				Gabi v8.7
kulczycka	22	anhydrite, H3PO4	D				-
Alipanah	23	REOs	E				-
Rabbani	24	-	-	X		X	EI
Chowdhury	25	Iron oxide	E			X	EI 3.7
Jin	26	-	-				EI 2.0
Li	27	Silver, copper, mREO	E			X	EI 3
Sanchez Moran	28	Iron salt	E, M			X	EI
Van Nielen	29	-	-		X	X	EI 3.8, cutoff
Liu	30	mercury	M, D			X	EI 3.5
Thompson	31	Eliminated waste disosal	-			X	EI 3.0
Magrini	32	-	E, M			X	EI 3.4

3 Compiled TEA data

Table S5: General information about the TEA studies reviewed. Some studies have multiple rows because they consider multiple scenarios.

Source	Unique Information	Feedstock	Capacity	Capacity unit	Capacity	Capacity unit	REO recovery (% mass)
31	bioleaching	FCC	18838	ton FCC catalyst feedstock/year	136.9523	mt REO/year	28-56
28	acid-free dissolution	HDD	342.42	tonnes of HDD shreds/year	2.53	tonnes didymium oxide/year	75
33		NiMH	2000	tonnes/year of used battery powder	244.6	t REO concentrate/year	85.13
34		Cell phone	2000	tonnes/year of cell phone waste	31.71	t/yr REO concentrate	78
32	bioleaching	NdFeB	0.33-1.2	tonnes/year of NIB magnets	0.095-0.35	kg/year REO	83
25	acid-free dissolution	NdFeB	100	tonnes/year NdFeB magnet sward	32	tonnes REO/year	97
35	metal distillation	NdFeB	744.6	kg of mischmetal (Nd70)	215.934	tonne/year Misch and Dy metal	60-100
36	supercritical fluid extraction	NdFeB	1228-2266	tonne/year preprocessed magnet	302-689	tonne/year iREO	97
36	supercritical fluid extraction	lamp phosphors	2606-10987	tonne/year preprocessed lamp phosphors	421-2410	tonne/year iREO	66
37	magnetic nanoparticles	geothermal brine	1344.375	tonne/year brine	2.151	tonne/year Eu	90
38	detailed SX process for iREO recovery	monazite	16000	tonne/year monazite concentrate	7291	tonne iREO/year	88.5
38		monazite	16000	tonne/year monazite concentrate	7114	tonne iREO/year	87.2
38		monazite	16000	tonne/year monazite concentrate	7412	tonne iREO/year	90
23	biosorption using Si-sol gel	coal fly ash	200000	tonnes/year CFA	47.1	tonnes mREO/year	70
23		lignite	200000	tonnes/year lignite	83	tonnes mREO/year	75
39	supercritical fluid extraction	coal fly ash	3650-4867	tons/year coal ash	1.1-2.69	tons REE/year	45-60
40		AMD	4397394	mt/year acidic mine wastewater	27	mt/year mREO	80
41		AMDp (Sterrett)	748.7493	tonne AMDp/year	1	tonne/year REE	80
41		AMDp (Woodlands)	1133.571	tonne AMDp/year	1	tonne/year REE	80
42	modelled SX, network sourcing strategy	AMDp	53000	tonnes AMD (preconcntrate)/year	444	tonnes REE/year	93
42		AMDp	53000	tonnes AMD (preconcntrate)/year	290	tonnes REE/year	61
42		AMDp	53000	tonnes AMD (preconcntrate)/year	290	tonnes REE/year	61
42		AMDp	53000	tonnes AMD (preconcntrate)/year	290	tonnes REE/year	61
42		AMDp	53000	tonnes AMD (preconcntrate)/year	212	tonnes REE/year	44
43		PA sludge	453000	tonnes PA-sludge/year	138	ton REO/year	48
43		PA sludge	453000	tonnes PA-sludge/year	49	ton REO/year	17

Table S6: System costs (CAPEX and OPEX), revenues, and profitability as payback period (PBP), return on investment (ROI), net present value (NPV), and internal rate of return (IRR). Information not provided in a paper is listed as a "-" in the table.

Source	Revenue (M$/year)	CAPEX (M$)	OPEX (M$/year)	PBP	ROI (%)	NPV (M$)	IRR (%)
[31]	3.88	1.27	1.76	-	-	5.78	44
[28]	0.38	0.268	0.22	-	-	-	-
[33]	33.7	26.92	13.88	1.58	63	95.9	46.1
[34]	77.2	53.2	18.23	1.09	91.61	296	60.9
[32]	0.031	-	0.11	-	-	-	-
[25]	1.24-2.69	0.48-0.91	0.61	-	-	-	-
[35]	23.8	1.62	5.63	-	-	299	143
[36]	17-65	13.8	13-22	0.5	-	17.5	16
[36]	7.0-58	14.8-15.8	8.0-23	0.5	-	128.3	49
[37]	2.2	6.8	1	-	-	-	18.1
[38]	131	90	88	4.5	-	135	-
[38]	104	90	99	-	-	-15	-
[38]	113.8	88	101	-	-	-62	-
[23]	0.75	1.4	76.69	-	-	-	-
[23]	28.55	1.2	24.40	-	-	28	-
[39]	0.77-2.37	0.4-0.5	1.3-3.5	-	-	-	-
[40]	0.783	3.835	5.33	-	-	-	-
[41]	0.3	22	3.4	-	-	-	-
[41]	0.6	16.6	5.7	-	-	-	-
[42]	49.42-70.46	186	21.4	-	-	(-80)-270	0-32
[42]	35.26-56.07	131	17.5	-	-	(-105)-110	(-15)-24
[42]	20.25-33.39	149	18.5	-	-	(-100)-160	(-12)-27
[42]	20.25-33.39	155	20.5	-	-	(-60)-275	2.-37
[42]	49.42-79.83	142	20.5	-	-	(-80)-190	(-60)-31
[43]	122	7.08	9.8	-	-	442	
[43]	112	7.55	27.1	-	-	179	

Table S7: The discounted cash flow analysis parameters for the reviewed TEA studies. Information not provided in a paper is listed as a "-" in the table. Depreciation methods are straight line (SL), double declining balance (DDB), modified accelerated cost recovery system (MACRS), and declining balance with a depreciation rate of 150% (150% DB).

Source	Discount rate (%)	Plant life (yr)	Operating time (hrs/yr)	Loan (yr)	Loan interest rate (%)	Depreciation period (yr)	Depreciation method	Income tax rate (%)	Startup time (months)	Construction (yr)	Cost basis
[31]	8	30	8000	10	8	10	DDB + SL	35	6	no	-
[28]	10	20	7884	10	7.5	7	DDB	39	no	no	2022
[33]	7	15	2000	10	9	10	SL	25	4	2.5	-
[34]	9	15	2000	10	9	10	SL	25	4	2.5	-
[32]	-	-	-	-	-	10	SL	30	-	-	-
[25]	-	20	2680	-	-	-	-	-	-	-	-
[35]	3.03	30	7446	15	4.5	20	DDB	35	0	3	2012
[36]	7.5	40	7446	-	-	-	-	-	0	2	2023
	7.5	40	7446	-	-	-	-	-	0	2	2023
[37]	10	30	8322	10	4	20	150% DB	38	0	2	2018
[38]	10	20	8000	no	no	20	SL	30	reduced productivity in first two years (80%,90%)	0	2020
[23]	8	20	8000	10	8	7, 15, or 19	MACRS	27	6	0	2020
[39]	-	15	7300	-	-	-	-	-	-	-	-
[40]	-	10	8760	-	-	-	-	-	-	-	-
[41]	-	25	8000	-	-	-	-	-	-	-	-
[42]	10	20	-	10	6	20	150% DB	26	0	3	2020-2021
[43]	5	10	-	0	0	10	SL	35	0	2	2023

4 Functional Unit Conversion Method

To convert between functional units of mREE, sREO, and sREM, we developed a method that uses literature data from LCAs of conventional REE production. We compiled data from many references to identify the amount of impact from individual process sections (Figure 6e and Figure 2).[2,3,7,9,10,13,17] The data for the REE recovery after further processing of mREEs (Figure 6d) was from one publication[7], as the LCI of other publications were less transparent. The REE content of REO compounds is provided in Table S8 and can be used to determine fractional REE content values dictated by REE composition. These sets of data provide the basis for the proposed conversion method.

Table S8: The REE content of various REO compounds.

REO	REE Atomic Weight (g/mol)	Mass REO	Mass O	REE Content of REO (wt %)	Deviation from Average REE Content (%)
Sc_2O_3	45	138	90	0.65	23
Y_2O_3	89	226	178	0.79	7
La_2O_3	139	326	278	0.85	1
Ce_2O_3	140	328	280	0.85	1
CeO_2	140	172	140	0.81	4
Pr_2O_3	141	330	282	0.85	1
Pr_6O_{11}	141	1021	845	0.83	2
Nd_2O_3	144	336	288	0.86	1
Pm_2O_3	145	338	290	0.86	1
Sm_2O_3	150	349	301	0.86	2
Eu_2O_3	152	352	304	0.86	2
Gd_2O_3	157	363	315	0.87	3
Tb_2O_3	159	366	318	0.87	3
Tb_4O_7	159	748	636	0.85	0
Dy_2O_3	163	373	325	0.87	3
Ho_2O_3	165	378	330	0.87	3
Er_2O_3	167	383	335	0.87	3
Tm_2O_3	169	386	338	0.88	3
Yb_2O_3	173	394	346	0.88	4
Lu_2O_3	175	398	350	0.88	4
Average				0.85	

Further, we want to highlight some limitations of this method. This method assumes separation and refining sections of novel systems will perform similarly to conventional systems with different feedstock mREO compositions. Therefore, systems with highly dissimilar compositions of mREE may be less accurate. Further, if a novel system has significantly lower impact in mining, beneficiation, leaching, and concentration sections (the blue bar), the contribution of separation and refining would be larger than conventional routes, especially BO and SP routes. The MW route would be the best choice for systems with low impacts from mREE production. For these three routes, the contributions for are provided in Figure 2b. Another source of inaccuracy is the REE content variable. For the REE content variable, we considered the common oxides formed by REEs and averaged the fractional REE content. This number is within 5% for all

REOs except Y_2O_3 (7% deviation) and Sc_2O_3 (23% deviation). Hence, if a product consists mainly of Sc, a fractional REE content closer to Sc_2O_3 (65.2%) may be appropriate.

The most accurate conversion between functional units would to directly add the impact of the separation or refining process sections (using whichever technology) to the mREE impact value (for whichever impact category). However, studies use a wide variety of life cycle impact assessment methods (LCIA), with different units and ways to quantify impact, that make it impossible to add the impact from further processing to the impact of mREE production. In the future, if LCAs of conventional REE production are harmonized, it would be possible to report impacts (with uncertainty) across multiple LCIA methods to enable this alternative method. For now, the proposed method above is suitable to enable rapid comparison of environmental impacts between systems to enhance decision-making.

5 Equations for calculating profitability

The following equations were to calculate the profitability, as return on investment (%) and cash flow (M$), using data from the reviewed studies. The calculated profitability was then used for comparisons in Figure 5 of the main text. We also provide the equation for calculating basket price that is used for Table 1, where P_{REO} is the price of each REO and f_{REO} is the fractional abundance by mass of that REO in the product. Prices and fractions of each REO were obtained individually from each reference and were not normalized across studies in this review.

$$\text{Basket Price} = \sum_{REO} P_{REO} \cdot f_{REO}$$

$$\text{Cash Flow} = \text{Annualized Revenue} - \text{Annualized CAPEX} - \text{OPEX}$$

$$\text{Return on Investment (ROI)} = \frac{\text{(Annualized revenue - Annualized OPEX)}}{\text{CAPEX}} \times 100$$

6 References

(1) Bullen, J. C.; Saleesongsom, S.; Gallagher, K.; Weiss, D. J. A Revised Pseudo-Second-Order Kinetic Model for Adsorption, Sensitive to Changes in Adsorbate and Adsorbent Concentrations. *Langmuir* **2021**, *37* (10), 3189–3201. https://doi.org/10.1021/acs.langmuir.1c00142.

(2) Arshi, P. S.; Vahidi, E.; Zhao, F. Behind the Scenes of Clean Energy: The Environmental Footprint of Rare Earth Products. *ACS Sustainable Chem. Eng.* **2018**, *6* (3), 3311–3320. https://doi.org/10.1021/acssuschemeng.7b03484.

(3) Bailey, G.; Joyce, P. J.; Schrijvers, D.; Schulze, R.; Sylvestre, A. M.; Sprecher, B.; Vahidi, E.; Dewulf, W.; Van Acker, K. Review and New Life Cycle Assessment for Rare Earth Production from Bastnäsite, Ion Adsorption Clays and Lateritic Monazite. *Resources, Conservation and Recycling* **2020**, *155*, 104675. https://doi.org/10.1016/j.resconrec.2019.104675.

(4) Marx, J.; Schreiber, A.; Zapp, P.; Walachowicz, F. Comparative Life Cycle Assessment of NdFeB Permanent Magnet Production from Different Rare Earth Deposits. *ACS Sustainable Chem. Eng.* **2018**, *6* (5), 5858–5867. https://doi.org/10.1021/acssuschemeng.7b04165.

(5) Zaimes, G. G.; Hubler, B. J.; Wang, S.; Khanna, V. Environmental Life Cycle Perspective on Rare Earth Oxide Production. *ACS Sustainable Chem. Eng.* **2015**, *3* (2), 237–244. https://doi.org/10.1021/sc500573b.

(6) Deng, H.; Kendall, A. Life Cycle Assessment with Primary Data on Heavy Rare Earth Oxides from Ion-Adsorption Clays. *Int J Life Cycle Assess* **2019**, *24* (9), 1643–1652. https://doi.org/10.1007/s11367-019-01582-1.

(7) Lee, J. C. K.; Wen, Z. Rare Earths from Mines to Metals: Comparing Environmental Impacts from China's Main Production Pathways. *Journal of Industrial Ecology* **2017**, *21* (5), 1277–1290. https://doi.org/10.1111/jiec.12491.

(8) Lee, J. C. K.; Wen, Z. Pathways for Greening the Supply of Rare Earth Elements in China. *Nat Sustain* **2018**, *1* (10), 598–605. https://doi.org/10.1038/s41893-018-0154-5.

(9) Zapp, P.; Marx, J.; Schreiber, A.; Friedrich, B.; Voßenkaul, D. Comparison of Dysprosium Production from Different Resources by Life Cycle Assessment. *Resources, Conservation and Recycling* **2018**, *130*, 248–259. https://doi.org/10.1016/j.resconrec.2017.12.006.

(10) Zapp, P.; Schreiber, A.; Marx, J.; Kuckshinrichs, W. Environmental Impacts of Rare Earth Production. *MRS Bulletin* **2022**, *47* (3), 267–275. https://doi.org/10.1557/s43577-022-00286-6.

(11) Vahidi, E.; Navarro, J.; Zhao, F. An Initial Life Cycle Assessment of Rare Earth Oxides Production from Ion-Adsorption Clays. *Resources, Conservation and Recycling* **2016**, *113*, 1–11. https://doi.org/10.1016/j.resconrec.2016.05.006.

(12) Vahidi, E.; Zhao, F. Environmental Life Cycle Assessment on the Separation of Rare Earth Oxides through Solvent Extraction. *Journal of Environmental Management* **2017**, *203*, 255–263. https://doi.org/10.1016/j.jenvman.2017.07.076.

(13) Vahidi, E.; Zhao, F. Assessing the Environmental Footprint of the Production of Rare Earth Metals and Alloys via Molten Salt Electrolysis. *Resources, Conservation and Recycling* **2018**, *139*, 178–187. https://doi.org/10.1016/j.resconrec.2018.08.010.

(14) Schreiber, A.; Marx, J.; Zapp, P.; Hake, J.-F.; Voßenkaul, D.; Friedrich, B. Environmental Impacts of Rare Earth Mining and Separation Based on Eudialyte: A New European Way. *Resources* **2016**, *5* (4), 32. https://doi.org/10.3390/resources5040032.

(15) Schreiber, A.; Marx, J.; Zapp, P. Life Cycle Assessment Studies of Rare Earths Production - Findings from a Systematic Review. *Science of The Total Environment* **2021**, *791*, 148257. https://doi.org/10.1016/j.scitotenv.2021.148257.

(16) Koltun, P.; Tharumarajah, A. Life Cycle Impact of Rare Earth Elements. *International Scholarly Research Notices* **2014**, *2014* (1), 907536. https://doi.org/10.1155/2014/907536.

(17) Koltun, P.; Klymenko, V. Cradle-to-Gate Life Cycle Assessment of the Production of Separated Mix of Rare Earth Oxides Based on Australian Production Route. **2020**. https://doi.org/10.33271/mining14.02.001.

(18) Schulze, R.; Lartigue-Peyrou, F.; Ding, J.; Schebek, L.; Buchert, M. Developing a Life Cycle Inventory for Rare Earth Oxides from Ion-Adsorption Deposits: Key Impacts and Further Research Needs. *J. Sustain. Metall.* **2017**, *3* (4), 753–771. https://doi.org/10.1007/s40831-017-0139-z.

(19) Sprecher, B.; Xiao, Y.; Walton, A.; Speight, J.; Harris, R.; Kleijn, R.; Visser, G.; Kramer, G. J. Life Cycle Inventory of the Production of Rare Earths and the Subsequent Production of NdFeB Rare Earth Permanent Magnets. *Environ. Sci. Technol.* **2014**, *48* (7), 3951–3958. https://doi.org/10.1021/es404596q.

(20) Brown, D.; Zhou, R.; Sadan, M. Critical Minerals and Rare Earth Elements in a Planetary Just Transition: An Interdisciplinary Perspective. *The Extractive Industries and Society* **2024**, *19*, 101510. https://doi.org/10.1016/j.exis.2024.101510.
(21) Ippolito, N. M.; Amato, A.; Innocenzi, V.; Ferella, F.; Zueva, S.; Beolchini, F.; Vegliò, F. Integrating Life Cycle Assessment and Life Cycle Costing of Fluorescent Spent Lamps Recycling by Hydrometallurgical Processes Aimed at the Rare Earths Recovery. *Journal of Environmental Chemical Engineering* **2022**, *10* (1), 107064. https://doi.org/10.1016/j.jece.2021.107064.
(22) Kulczycka, J.; Kowalski, Z.; Smol, M.; Wirth, H. Evaluation of the Recovery of Rare Earth Elements (REE) from Phosphogypsum Waste – Case Study of the WIZÓW Chemical Plant (Poland). *Journal of Cleaner Production* **2016**, *113*, 345–354. https://doi.org/10.1016/j.jclepro.2015.11.039.
(23) Alipanah, M.; Park, D. M.; Middleton, A.; Dong, Z.; Hsu-Kim, H.; Jiao, Y.; Jin, H. Techno-Economic and Life Cycle Assessments for Sustainable Rare Earth Recovery from Coal Byproducts Using Biosorption. *ACS Sustainable Chem. Eng.* **2020**, *8* (49), 17914–17922. https://doi.org/10.1021/acssuschemeng.0c04415.
(24) Rabbani, M.; Werner, J.; Fahimi, A.; Vahidi, E. Innovative Pilot-Scale Process for Sustainable Rare Earth Oxide Production from Coal Byproducts: A Comprehensive Environmental Impact Assessment. *Journal of Rare Earths* **2024**. https://doi.org/10.1016/j.jre.2024.04.004.
(25) Chowdhury, N. A.; Deng, S.; Jin, H.; Prodius, D.; Sutherland, J. W.; Nlebedim, I. C. Sustainable Recycling of Rare-Earth Elements from NdFeB Magnet Swarf: Techno-Economic and Environmental Perspectives. *ACS Sustainable Chem. Eng.* **2021**, *9* (47), 15915–15924. https://doi.org/10.1021/acssuschemeng.1c05965.
(26) Jin, H.; Afiuny, P.; McIntyre, T.; Yih, Y.; Sutherland, J. W. Comparative Life Cycle Assessment of NdFeB Magnets: Virgin Production versus Magnet-to-Magnet Recycling. *Procedia CIRP* **2016**, *48*, 45–50. https://doi.org/10.1016/j.procir.2016.03.013.
(27) Li, Z.; Diaz, L. A.; Yang, Z.; Jin, H.; Lister, T. E.; Vahidi, E.; Zhao, F. Comparative Life Cycle Analysis for Value Recovery of Precious Metals and Rare Earth Elements from Electronic Waste. *Resources, Conservation and Recycling* **2019**, *149*, 20–30. https://doi.org/10.1016/j.resconrec.2019.05.025.
(28) Sanchez Moran, E.; Prodius, D.; Nlebedim, I. C.; Mba Wright, M. Rare-Earth Elements Recovery from Electronic Waste: Techno-Economic and Life Cycle Analysis. *ACS Sustainable Chem. Eng.* **2024**, *12* (38), 14164–14172. https://doi.org/10.1021/acssuschemeng.4c04100.
(29) van Nielen, S. S.; Miranda Xicotencatl, B.; Tukker, A.; Kleijn, R. Ex-Ante LCA of Magnet Recycling: Progressing towards Sustainable Industrial-Scale Technology. *Journal of Cleaner Production* **2024**, *458*, 142453. https://doi.org/10.1016/j.jclepro.2024.142453.
(30) Liu, L.; Keoleian, G. A. LCA of Rare Earth and Critical Metal Recovery and Replacement Decisions for Commercial Lighting Waste Management. *Resources, Conservation and Recycling* **2020**, *159*, 104846. https://doi.org/10.1016/j.resconrec.2020.104846.
(31) Thompson, V. S.; Gupta, M.; Jin, H.; Vahidi, E.; Yim, M.; Jindra, M. A.; Nguyen, V.; Fujita, Y.; Sutherland, J. W.; Jiao, Y.; Reed, D. W. Techno-Economic and Life Cycle Analysis for Bioleaching Rare-Earth Elements from Waste Materials. *ACS Sustainable Chem. Eng.* **2018**, *6* (2), 1602–1609. https://doi.org/10.1021/acssuschemeng.7b02771.
(32) Magrini, C.; Jagodzińska, K. Can Bioleaching of NIB Magnets Be an Answer to the Criticality of Rare Earths? An Ex-Ante Life Cycle Assessment and Material Flow Cost Accounting. *Journal of Cleaner Production* **2022**, *365*, 132672. https://doi.org/10.1016/j.jclepro.2022.132672.
(33) Otron, A. M. A.-A.; Tran, L.-H.; Blais, J.-F. Mass Balance and Economic Study of a Treatment Chain for Nickel, Cobalt and Rare Earth Elements Recovery from Ni-MH Batteries. *Environmental Technology 0* (0), 1–15. https://doi.org/10.1080/09593330.2024.2387374.
(34) Diallo, S.; Tran, L.-H.; Larivière, D.; Blais, J.-F. Mass Balance and Economic Study of a Treatment Chain for Rare Earths, Base Metals and Precious Metals Recovery from Used Smartphones. *Minerals Engineering* **2024**, *215*, 108824. https://doi.org/10.1016/j.mineng.2024.108824.
(35) Chinwego, C.; Wagner, H.; Giancola, E.; Jironvil, J.; Powell, A. Technoeconomic Analysis of Rare-Earth Metal Recycling Using Efficient Metal Distillation. *JOM* **2022**, *74* (4), 1296–1305. https://doi.org/10.1007/s11837-021-05045-7.
(36) Azimi, G.; Sauber, M. E.; Zhang, J. Technoeconomic Analysis of Supercritical Fluid Extraction Process for Recycling Rare Earth Elements from Neodymium Iron Boron Magnets and Fluorescent Lamp Phosphors. *Journal of Cleaner Production* **2023**, *422*, 138526. https://doi.org/10.1016/j.jclepro.2023.138526.

(37) Liu, S.; Featherston, E. R.; Cotruvo, J. A.; Baiz, C. R. Lanthanide-Dependent Coordination Interactions in Lanmodulin: A 2D IR and Molecular Dynamics Simulations Study. *Phys. Chem. Chem. Phys.* **2021**, *23* (38), 21690–21700. https://doi.org/10.1039/D1CP03628A.

(38) Mukhlis, R. Z.; Lee, J.-Y.; Kang, H. N.; Haque, N.; Pownceby, M. I.; Bruckard, W. J.; Rhamdhani, M. A.; Jyothi, R. K. Techno-Economic Evaluation of an Environmental-Friendly Processing Route to Extract Rare Earth Elements from Monazite. *Cleaner Engineering and Technology* **2024**, *20*, 100742. https://doi.org/10.1016/j.clet.2024.100742.

(39) Das, S.; Gaustad, G.; Sekar, A.; Williams, E. Techno-Economic Analysis of Supercritical Extraction of Rare Earth Elements from Coal Ash. *Journal of Cleaner Production* **2018**, *189*, 539–551. https://doi.org/10.1016/j.jclepro.2018.03.252.

(40) Zhang, W.; Honaker, R. Q. Rare Earth Elements Recovery Using Staged Precipitation from a Leachate Generated from Coarse Coal Refuse. *International Journal of Coal Geology* **2018**, *195*, 189–199. https://doi.org/10.1016/j.coal.2018.06.008.

(41) Fritz, A. G.; Tarka, T. J.; Mauter, M. S. Technoeconomic Assessment of a Sequential Step-Leaching Process for Rare Earth Element Extraction from Acid Mine Drainage Precipitates. *ACS Sustainable Chem. Eng.* **2021**, *9* (28), 9308–9316. https://doi.org/10.1021/acssuschemeng.1c02069.

(42) Larochelle, T.; Noble, A.; Ziemkiewicz, P.; Hoffman, D.; Constant, J. A Fundamental Economic Assessment of Recovering Rare Earth Elements and Critical Minerals from Acid Mine Drainage Using a Network Sourcing Strategy. *Minerals* **2021**, *11* (11), 1298. https://doi.org/10.3390/min11111298.

(43) Jang, G. G.; Thompson, J. A.; Meyer, P. A.; Zhang, P.; Shen, Z.; Tsouris, C. Technoeconomic Assessment of Phosphoric Acid and Rare Earth Element Recovery from Phosphoric Acid Sludge. *Sustainability* **2024**, *16* (16), 6984. https://doi.org/10.3390/su16166984.
